# Extensive protein pyrophosphorylation revealed in human cell lines

**DOI:** 10.1038/s41589-024-01613-5

**Published:** 2024-04-25

**Authors:** Jeremy A. M. Morgan, Arpita Singh, Leonie Kurz, Michal Nadler-Holly, Max Ruwolt, Shubhra Ganguli, Sheenam Sharma, Martin Penkert, Eberhard Krause, Fan Liu, Rashna Bhandari, Dorothea Fiedler

**Affiliations:** 1 Leibniz-Forschungsinstitut für Molekulare Pharmakologie (FMP), Berlin, Germany; 2 Laboratory of Cell Signalling, Centre for DNA Fingerprinting and Diagnostics, Hyderabad, India; 3 Graduate Studies, Regional Centre for Biotechnology, Faridabad, India; 4 Institute of Chemistry, Humboldt-Universität zu Berlin, Berlin, Germany

**Keywords:** Mass spectrometry, Proteomics, Post-translational modifications, Transcription, Kinases

## Abstract

Reversible protein phosphorylation is a central signaling mechanism in eukaryotes. Although mass-spectrometry-based phosphoproteomics has become routine, identification of non-canonical phosphorylation has remained a challenge. Here we report a tailored workflow to detect and reliably assign protein pyrophosphorylation in two human cell lines, providing, to our knowledge, the first direct evidence of endogenous protein pyrophosphorylation. We manually validated 148 pyrophosphosites across 71 human proteins, the most heavily pyrophosphorylated of which were the nucleolar proteins NOLC1 and TCOF1. Detection was consistent with previous biochemical evidence relating the installation of the modification to inositol pyrophosphates (PP-InsPs). When the biosynthesis of PP-InsPs was perturbed, proteins expressed in this background exhibited no signs of pyrophosphorylation. Disruption of PP-InsP biosynthesis also significantly reduced rDNA transcription, potentially by lowering pyrophosphorylation on regulatory proteins NOLC1, TCOF1 and UBF1. Overall, protein pyrophosphorylation emerges as an archetype of non-canonical phosphorylation and should be considered in future phosphoproteomic analyses.

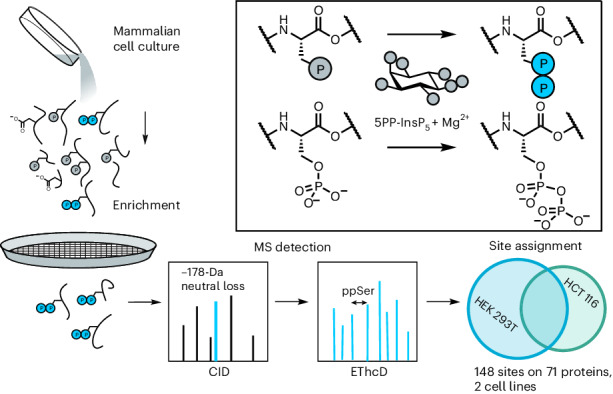

## Abstract (web summary)

A tailored proteomics workflow to identify endogenous protein pyrophosphorylation in human cells was developed, revealing the dependence of the modification on inositol pyrophosphates and a putative function in rDNA transcription.

## Main

**Fig. 1:**
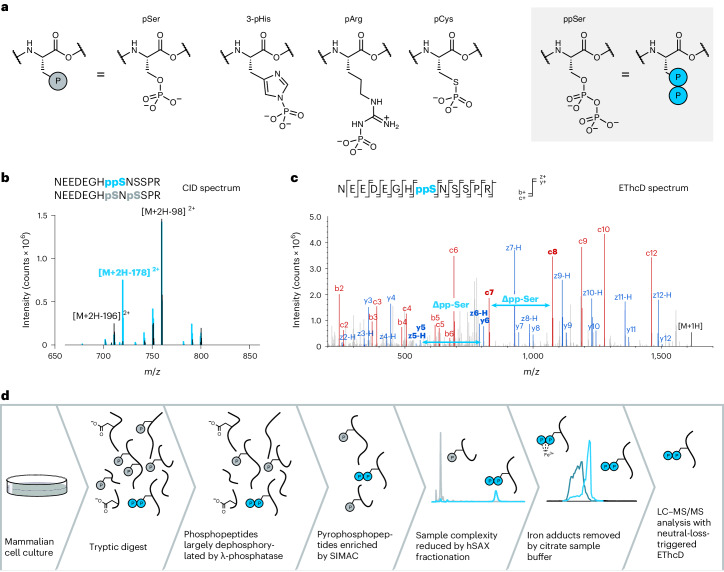
Enrichment and detection of pyrophosphorylated peptides using mass spectrometry. **a**, Examples of protein phosphorylation on different amino acid side chains. Canonical phosphorylation sites include phosphoserine (pSer), whereas non-canonical sites are exemplified by phosphohistidine (3-pHis), phosphoarginine (pArg), phosphocysteine (pCys) and pyrophosphoserine (ppSer). **b**, Fragmentation via CID of a pyrophosphopeptide results in a characteristic neutral loss (−178 Da) compared to the corresponding bisphosphopeptide. **c**, Fragmentation of a pyrophosphopeptide using electron transfer dissociation combined with EThcD shows excellent sequence coverage while leaving the modification intact. **d**, Development of a sample preparation workflow, tailored to the enrichment and subsequent detection of tryptic pyrophosphopeptides.

The specific phosphorylation of proteins is a fundamental mechanism of intracellular signal transduction across the domains of life^1,2^. In humans, kinases and phosphatases dedicated to the writing and erasing of protein phosphorylation make up almost 2.5% of the genome^3–5^. After the discovery of serine (Fig. 1a), threonine and tyrosine phosphorylation through biochemical approaches, mass spectrometry (MS)-based proteomics became the primary method to investigate the function and regulation of canonical Ser/Thr/Tyr phosphorylation^6^. As of 2024, more than 290,000 phosphorylation sites have been reported in the PhosphoSitePlus database, identified almost entirely by phosphoproteomic approaches^7^.

In contrast, phosphorylation of non-canonical amino acid residues in humans (histidine, arginine, cysteine, aspartate, glutamate and lysine; Fig. 1a) has been established with varying success. Adapting traditional bottom-up phosphoproteomics workflows to allow for high-throughput detection of these modifications has proven challenging, as the low pH conditions used in sample preparation typically led to the hydrolysis of the acid-labile phosphoramidate, thiophosphate and acylphosphate moieties^8,9^. Rapid acidic enrichment employed at low temperatures has, however, proven successful for phosphohistidine and phosphoarginine detection in bacterial backgrounds^10,11^. There is also an additional burden on the spectral interpretation, as convincing assignment of a novel non-canonical site requires excluding a misassigned canonical site. To improve assignment accuracy, methods have incorporated characteristic neutral loss patterns^12^ and immonium ion formation^10^ into the detection and assignment process. In human backgrounds, identification of phosphohistidine (pHis) is arguably the most advanced, with 14 sites biochemically validated^13^ and several hundred sites identified across different phosphoproteomics studies. However, there are still considerable concerns regarding the validity of these high-throughput site assignments^14^.

Protein pyrophosphorylation, the phosphorylation of a phosphoserine (pSer) residue to yield pyrophosphoserine (ppSer; Fig. 1a), is an additional non-canonical phosphorylation that is often overlooked^15–17^. This non-enzymatic posttranslational modification is mediated by high-energy inositol pyrophosphate messengers (PP-InsPs), which can transfer their β-phosphoryl group to protein substrates in the presence of Mg^2+^ ions^16^. Pyrophosphorylation was established using radiolabeled 5-diphosphoinositol pentakisphosphate (5PP-InsP_5_), putatively the most abundant PP-InsP, demonstrating that a peptide or protein substrate can accept the radiolabel at only a pre-phosphorylated residue^16^. ppSer exhibits differences in stability compared to pSer, notably a resistance to hydrolysis by common protein phosphatases and λ-phosphatase.

The *Saccharomyces cerevisiae* proteins Nsr1, Srp40 and YGR130C were the first eukaryotic proteins shown to undergo pyrophosphorylation in vitro^15^. Subsequently, a handful of mammalian targets of in vitro protein pyrophosphorylation were identified, including nucleolar and coiled-body phosphoprotein 1 (NOLC1), Treacher Collins syndrome protein 1 (TCOF1), adaptor protein complex AP-3 subunit beta-1 (AP3B1), cytoplasmic dynein 1 intermediate chain 2 (DC1L2) and the oncoprotein MYC^16,18–20^. Indirect evidence of endogenous pyrophosphorylation has relied on a ‘back-phosphorylation’ assay, where potential targets are expressed and purified from a PP-InsP-rich cell line. In such a cell line, endogenous pyrophosphorylation should be elevated, and the subsequent in vitro phosphoryl transfer to this target should be decreased, when compared to the protein obtained from a cell line with low PP-InsP levels^21^. Although essential to discovery, these tools are low throughput and cannot provide direct information on the sites of modification.

Detection of endogenous pyrophosphorylation by mass spectrometry (MS)-based proteomics would address these limitations. Compared to other modes of non-canonical phosphorylation, pyrophosphorylation is relatively acid stable^15^, suggesting that traditional phosphoproteomics enrichment techniques should be compatible. However, the major technical challenge in enriching and detecting pyrophosphopeptides is their differentiation from peptides monophosphorylated at multiple positions. In particular, bisphosphorylated peptides (peptides containing two monophosphate groups) are isobaric with pyrophosphopeptides, making the observation of the molecular ion uninformative. An unambiguous assignment requires an inversion of the traditional probability-based assignment; the peptide must be assumed to be bisphosphorylated, unless pyrophosphorylation can be asserted.

Here we report the development of a dedicated pyrophosphoproteomics workflow for the detection and unambiguous assignment of endogenous pyrophosphorylation sites from mammalian cell lysates. Using neutral-loss-triggered electron transfer dissociation combined with higher-energy collision dissociation (EThcD) liquid chromatography–tandem mass spectrometry (LC–MS/MS) analysis, 108 and 78 sites were identified from HEK293T and HCT116 cell lines, respectively. Protein pyrophosphorylation sites predominantly occurred in acidic serine-rich stretches, and most of the identified pyrophosphoproteins localized to the nucleus and nucleolus. Several proteins with newly identified pyrophosphorylation sites also accepted radiolabeled phosphate from [β^32^P]5PP-InsP_5_ in vitro, supporting the non-enzymatic mechanism and the validity of both detection methods. In a functional readout for pyrophosphorylation of nucleolar proteins, we observed significantly impaired rDNA transcription in 5PP-InsP_5_-depleted cells. In sum, protein pyrophosphorylation can now be added unequivocally to the growing list of endogenous phosphorylation motifs in human cell lines.

## Results

### Establishment of a pyrophosphoproteomics workflow

**Extended Data Fig. 1:**
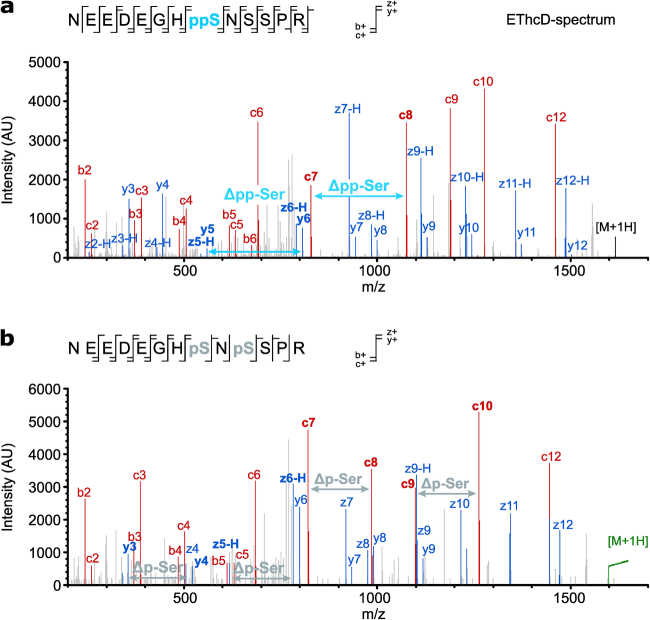
EThcD spectra of model peptides. **a**) EThcD fragmentation spectrum of the pyrophosphopeptide from Fig. 1c. **b**) EThcD fragmentation spectrum of the corresponding bisphosphopeptide. N-terminal fragments are labeled in red, C-terminal fragments in blue. Arrows indicate the characteristic fragments, which enable a distinction between pyrophosphorylated and bisphosphorylated peptide species in spite of their equal MS1 mass.

Using synthetic peptide standards, we previously established specific mass spectrometric detection of pyrophosphopeptides based on a characteristic neutral loss of –178 *m*/*z*, corresponding to the loss of pyrophosphoric acid (H_4_P_2_O_7_) during collision-induced dissociation (CID) fragmentation (Fig. 1b)^22^. This neutral loss did not occur in Ser/Thr/Tyr monophosphorylated or bisphosphorylated peptides. Detection of this neutral loss from CID fragmentation of the proteome could be used as a trigger during shotgun proteomic analysis to identify candidate pyrophosphopeptide parent ions, which then undergo selective EThcD fragmentation. The EThcD spectra provided excellent sequence coverage while the modification stayed intact, enabling assignment of pyrophosphorylation sites. After implementing further improvements, including an optimized neutral loss filter, fine-tuned fragmentation parameters and the exclusion of low-charge precursor ions, this method was again applied to model pyrophosphopeptides (Fig. 1c and Extended Data Fig. 1a) and bisphosphopeptides (Extended Data Fig. 1b). Consistent neutral loss detection and reproducible site assignment indicated that this triggered MS approach was suitable for the identification of pyrophosphorylation sites in cell lysates.

**Extended Data Fig. 2:**
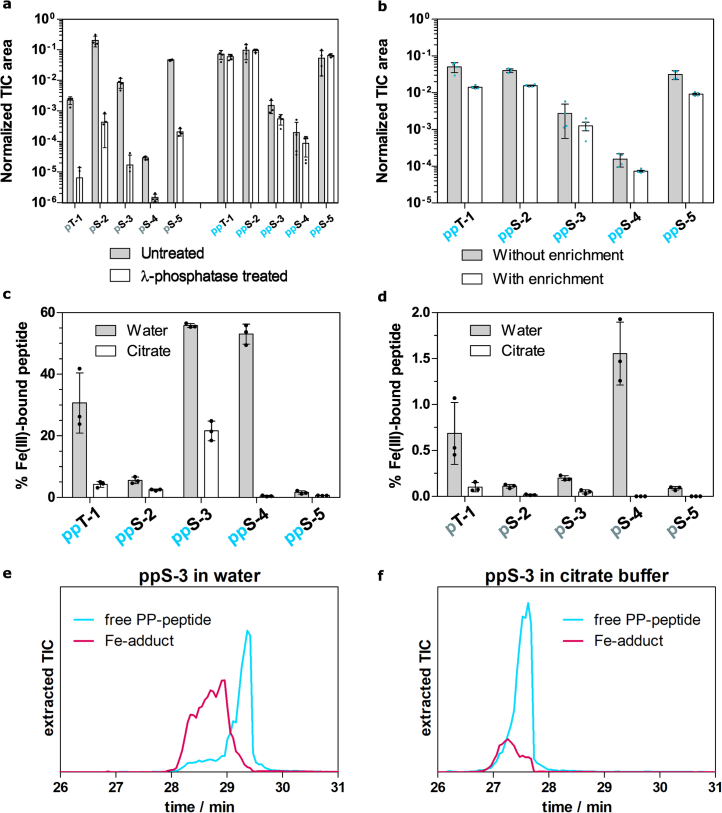
Validation of workflow steps. **a**) Pyrophosphopeptidepeptide stability to λ-phosphatase. Five synthetic pyrophosphopeptides and the corresponding monophosphopeptides were treated with the enzyme for 5 h. Data are presented as mean ± SD of four replicates. **b**) Showing retention of pyrophosphopeptides during SIMAC enrichment. Five synthetic pyrophosphopeptides were spiked into a background of HCT116 tryptic digest before or after enrichment and detected by LC-MS. Data are presented as mean ± SD of four replicates. **c**)-**f**) Improvement of LC-peak shape and reduction of Fe-adducts by citrate resuspension buffer (50 mM sodium citrate, 3% MeCN). **c**)/**d**) Proportion of Fe-adducts of synthetic pyrophosphopeptides (c) or monophosphopeptides (d) detected by LC-MS after sample resuspension in water vs. citrate buffer. Data are presented as mean ± SD of three replicates. **e**)/**f**) Representative total ion chromatograms (three replicates) of a synthetic pyrophosphopeptide in free or Fe^3+^-bound form after resuspension in water (e) or citrate buffer (f).

In first attempts, samples were prepared using a standard high pH fraction and immobilized metal ion affinity chromatography (IMAC) enrichment approach for phosphoproteomics^23^ and were subsequently analyzed using the triggered MS method. Unfortunately, although the spiked-in pyrophosphopeptide standards were reliably detected, only one endogenous site was identified. Therefore, a dedicated sample preparation workflow for enrichment of pyrophosphorylated tryptic peptides was developed (Fig. 1d). We tailored a standard phosphoproteomics workflow^24^ toward pyrophosphopeptide selection utilizing a set of synthetic pyrophosphopeptides (and the corresponding phosphopeptides) of varying sequence characteristics for optimization (Extended Data Fig. 2). Proteomic material was generated from HCT116 or HEK293T cells using standard protocols for tryptic digestion. To reduce competition with phosphopeptides during subsequent enrichment, the digested material was then treated with λ-phosphatase. As previously reported, this phosphatase hydrolyzes a large proportion of monophosphopeptides while leaving the pyrophosphoryl groups intact (Extended Data Fig. 2a)^16,25^. A standard sequential elution from immobilized metal ion affinity chromatography (SIMAC) enrichment, featuring an additional low pH washing step designed to elute acidic peptides, was then performed^26^. Pyrophosphopeptides were largely retained during SIMAC (Extended Data Fig. 2b), and the overall material mass was reduced more than 40-fold. This retention is likely supported by the lower predicted pKa value of the pyrophosphoryl moiety, in comparison to phosphoryl groups or acidic amino acid side chains.

Despite the phosphatase treatment and SIMAC enrichment, peptides with polyacidic amino acid stretches and multiple monophosphorylation sites were still abundant, so an offline fractionation step was implemented to further reduce sample complexity (Supplementary Fig. 1). An ultra-performance liquid chromatography (UPLC) hydrophilic SAX (hSAX)^27^ column using a quaternary ammonium stationary phase on a hydrophilic polymeric support was selected, owing to orthogonality with the low pH reverse-phase chromatography used in the LC–MS separation and the ability to separate analytes of differing negative charge and polarity^6^.

While establishing the workflow, we observed that pyrophosphorylated standard peptides were often detected in complex with Fe^3+^ ions during LC–MS analysis. Crucially, these adducts formed in the liquid phase, as evidenced by distinct retention times and peak shapes. Similar behavior was previously reported for highly phosphorylated peptides and could be resolved through the addition of metal chelating agents^28,29^. Therefore, sodium citrate (50 mM) was added into the sample resuspension buffer, which led to a substantial decrease of Fe^3+^ adduct formation (Extended Data Fig. 2c–f)^28,29^. Overall, the sample preparation workflow (Fig. 1d) now seemed adequate for the enrichment of pyrophosphopeptides from complex samples.

### Reliable annotation of endogenous pyrophosphorylation sites

**Fig. 2:**
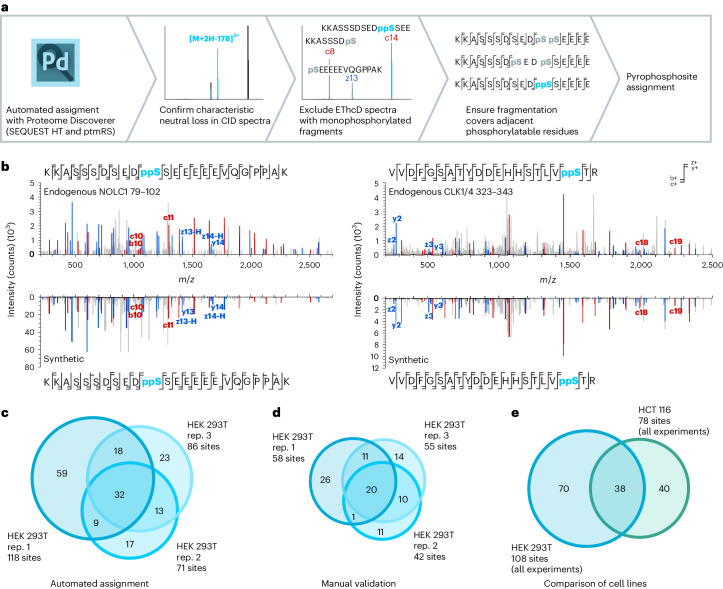
Assignment and validation of endogenous pyrophosphorylation sites. **a**, Workflow for the assignment of pyrophosphorylation sites after automated assignment with Proteome Discoverer. **b**, Comparison of EThcD spectra obtained from complex samples (top) with synthetically prepared pyrophosphopeptides (bottom). Fragment ions critical for the assignment of pyrophosphorylation sites are indicated in red (b-ion and c-ion series) or blue (y-ion and z-ion series). See detailed assignment in Supplementary Fig. 3. **c**, Venn diagram of the number of pyrophosphorylation sites detected by Proteome Discoverer in *n* = 3 biological replicates prepared from HEK293T cell lysates. **d**, Venn diagram of the number of pyrophosphorylation sites detected after manual assignment using *n* = 3 biological replicates from **c**. **e**, Overlap of pyrophosphorylation sites detected in HEK293T and HCT116 cells.

**Extended Data Fig. 3:**
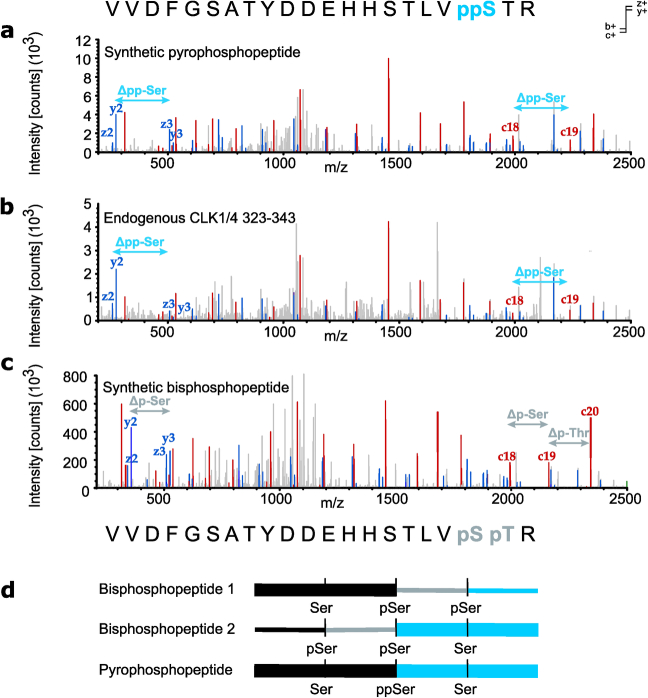
Fragmentation of pyrophospho- vs. bisphosphopeptides. **a**)-**c**) EThcD spectra of an endogenous pyrophosphopeptide (CLK1, residues 323-343) (**a**) and the corresponding synthetic pyrophospho- (**b**) and bisphosphopeptides (**c**), highlighting the fragment ions essential for distinguishing the two modifications. **d**) Schematic demonstrating how a mixture of bisphosphopeptides may contain all fragments typical of the corresponding pyrophosphopeptide and lead to false-positive assignment of a pyrophosphopeptide.

We next subjected the widely used mammalian cell line HEK293T to the pyrophosphoproteomics workflow. Using CID neutral-loss-triggered EThcD MS, many putative pyrophosphorylation sites were detected. To avoid incorrect assignment of multiply phosphorylated peptides (particularly bisphosphorylated peptides) as pyrophosphorylated, careful analysis of the data was required (Fig. 2a). Initial annotation was made on the basis of the SEQUEST HT engine with a fixed value peptide spectrum match (PSM) validator and ptmRS assignment as nodes in a Proteome Discoverer workflow. In the resulting dataset, some spectra could be directly assigned as pyrophosphopeptides based on unambiguous fragmentation, but many spectra were annotated both as pyrophosphorylated and as bisphosphorylated peptides with similar certainty. Increasing the threshold for the *P* value during automated assignment did not alleviate this problem. It became apparent that co-elution (and co-fragmentation) of bisphosphorylated peptides with pyrophosphopeptides could produce ambiguous mixed spectra: if two bisphosphopeptides with overlapping phosphorylation pairs are co-fragmented, all fragments required to annotate a pyrophosphorylation site to that central residue are present (Extended Data Fig. 3). This means that the interpretation of each spectrum must exclude the possibility of bisphosphopeptide mixtures before an assignment can be made. Therefore, a three-step protocol to assess automatically assigned pyrophosphorylation sites was established (Fig. 2a).

During MS analysis, each precursor ion is first fragmented by CID. In the synthetic pyrophosphopeptide standards, the −178-*m*/*z* (H_4_P_2_O_7_) neutral loss peak was consistently among the three most intense signals in the CID spectra, the other two being −98 *m*/*z* (H_3_PO_4_) and −196 *m*/*z* (H_6_P_2_O_8_). The HEK293T proteomic data showed that parent ions exhibiting weak −178-*m*/*z* neutral loss peaks during CID fragmentation were frequently assigned as ambiguous based on EThcD fragmentation. This is consistent with bisphosphopeptide co-fragmentation, because bisphosphopeptides can produce only −98-*m*/*z* and –196-*m*/*z* neutral losses, making these signals proportionally higher. Therefore, in the first assessment step, parent ions exhibiting a −178-*m*/*z* neutral loss with an intensity outside the three most intense signals were discarded, as they were likely bisphosphopeptide/pyrophosphopeptide mixtures (Supplementary Fig. 2).

In the second step, EThcD fragmentation spectra of candidate ions triggered by the detection of the neutral loss peak during CID were then manually examined using the Molecular Weight Calculator (https://github.com/PNNL-Comp-Mass-Spec/Molecular-Weight-Calculator-VB6)^30^ for evidence of peptide fragments containing a single phosphorylated residue—this is possible only if the peptide is bisphosphorylated. Again, candidate ions exhibiting such fragments were discarded (Fig. 2a and Supplementary Fig. 2).

Finally, in the third step, the extent of fragmentation across the putative pyrophosphorylation site was assessed. Missing fragments, particularly those encompassing a canonically phosphorylatable residue, such as serine or threonine, can lead to the misassignment of a bisphosphorylated peptide as pyrophosphorylated, and, as such, spectra with key fragments missing were discarded (Supplementary Fig. 2). The remaining spectra correspond to genuine pyrophosphorylation sites.

To validate the pyrophosphosite assignment, we synthesized pyrophosphopeptides based on two detected sequences: NOLC1 79–102 and CLK1/4 323–343 (Fig. 2b). Both peptide sequences contain phosphorylatable residues directly adjacent to the putative pyrophosphorylation sites. To prove that pyrophosphorylation is present, sequential ions consistent with the unphosphorylated peptide fragment immediately preceding the putative site (for example, ppNOLC1 79–88) and the sequential pyrophosphorylated peptide fragment containing the putative site (for example, ppNOLC1 79–89) must be detected simultaneously (in the absence of a singly phosphorylated peptide fragment indicative of bisphosphorylation). For the NOLC1 sequence, the c10 and c11 ion couplet and the z13-H and z14-H ion couplet in the c/z ions series were observed in the fragmentation spectra of both the synthetic peptide and the endogenous peptide, confirming the presence of the putative pyrophosphorylation site. Similarly, the CLK1/4 sequence exhibited the z2/z3 and c18/c19 ion couplets in the c/z ions series and the y2/y3 ion couplet in the b/y ion series, in both synthetic and endogenous peptide fragmentation spectra, consistent with pyrophosphorylation at Ser341. No fragments indicative of monophosphorylation were detected in any spectra, and, crucially, the diagnostic ion couplets were absent in the fragmentation spectra of the corresponding bisphosphopeptide (Extended Data Fig. 1). Together, these data validated our assignment approach for the reliable and correct identification of endogenous pyrophosphorylation sites.

**Extended Data Fig. 4:**
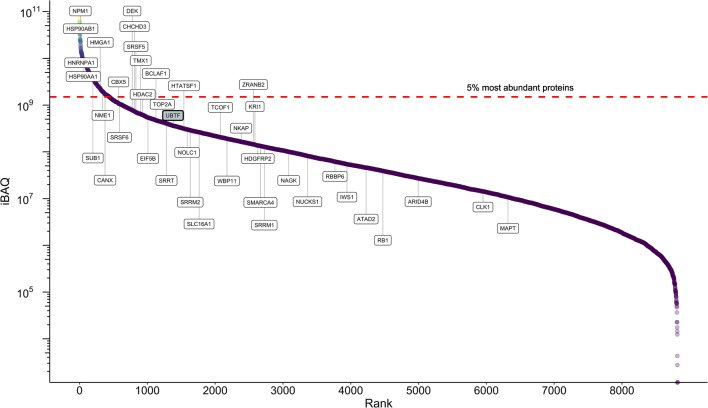
Expression levels of pyrophosphoproteins. iBAQ abundance scores of 8821 identified proteins from HEK293T cells (after removing decoys and contaminants) were plotted against their rank in the list of iBAQs. All identified pyrophosphoproteins from a HEK293T biological triplicate are highlighted in white boxes. UBF1, another substrate of pyrophosphorylation that was detected by radiolabeling, but not mass spectrometry, ranks among the more abundant proteins and is highlighted in grey.

After applying the pyrophosphoproteomics workflow and assignment strategy to HEK293T cell lysates, three biological replicates were analyzed. A total of 171 unique pyrophosphorylation sites were detected by automated assignment, 93 of which were manually validated (an average of 51 per replicate; Fig. 2c,d and Supplementary Table 1). Forty pyrophosphoproteins were identified from manually validated sites. Although a core of 20 manually validated sites was seen in all replicates, a significant portion of the sites (51) were exclusively observed in a single replicate. This is likely due to variation in the sample background of each replicate, differentially co-eluting and masking these low abundant species. Three proteins exhibiting multiple pyrophosphorylation sites were heavily overrepresented in the triplicate. NOLC1, TCOF1 and serine/arginine repetitive matrix protein 1 (SRRM1) sites were found in all replicates and represented 43 of the 93 total sites assigned. Overall, compared to the HEK293T proteome, the identified pyrophosphoproteins were expressed at intermediate to high levels, illustrating the challenge of sufficiently enriching pyrophosphopeptides (Extended Data Fig. 4).

After establishing the detectability of pyrophosphorylation, we reanalyzed the replicate 1 HEK293T dataset using a decoy searching approach with the target decoy PSM validator node in Proteome Discoverer. Of the 58 manually assigned sites, 19 were correctly assigned, and a further 34 sites discarded during manual evaluation were identified (Supplementary Table 2). Many of these additional sites were plausible but did not fulfil the criteria for unambiguous assignment, highlighting the utility of an automated decoy search in a preliminary assessment of proteome pyrophophosphorylation.

During method development, lysates from HEK293T and another human cell line, HCT116 colon cancer cells, were also frequently subjected to pyrophosphoproteomic analysis, and a total of 78 sites on 33 proteins were identified in HCT116 (Supplementary Table 3). Many of these pyrophosphorylation sites overlap with sites from HEK293T lysates (Fig. 2e), further corroborating the reliability of the assignment strategy.

### Pyrophosphorylation is commonly found on nucleolar proteins

**Fig. 3:**
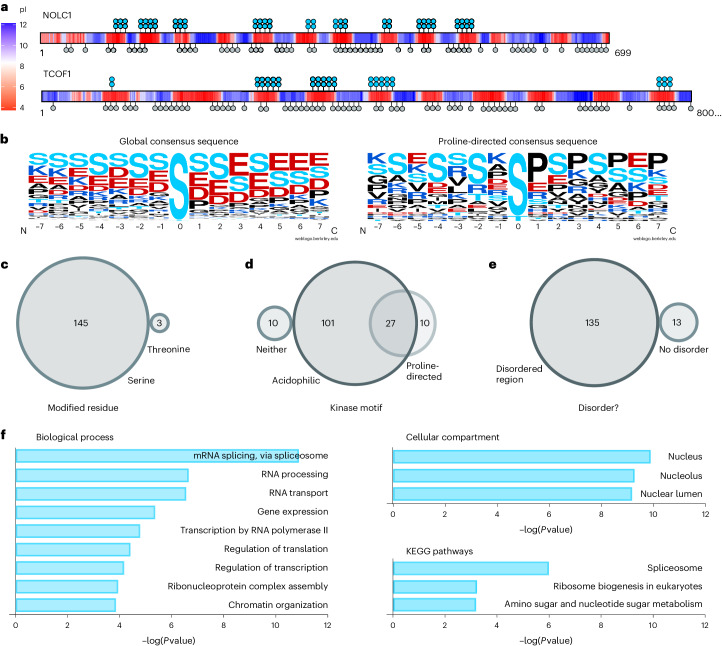
Properties of pyrophosphorylation sites. **a**, Illustration of the localization of pyrophosphorylation sites to acidic regions in NOLC1 and TCOF1. The local isoelectric point (pI) was calculated using a custom R script. ppSer residues are shown in turquoise, and previously reported phosphorylation sites are indicated in gray (PhosphoSitePlus (ref. ^7^)). **b**, Consensus sequence of all pyrophosphorylation sites detected (left) and consensus sequence after removal of CK2 consensus sites (right). Sequence logos were generated using WebLogo (ref. ^65^). **c**, Side chain of modification for all pyrophosphorylation sites. **d**, Kinase motifs surrounding the pyrophosphorylation sites. **e**, Analysis of order/disorder around pyrophosphorylation sites. **f**, Gene Ontology analysis of pyrophosphoproteins using Enrichr (Supplementary Table 4; https://maayanlab.cloud/Enrichr/). *P* values were obtained using Fisher’s exact test.

Two nucleolar proteins, NOLC1 and TCOF1, were heavily pyrophosphorylated, containing 34 and 18 different pyrophosphorylation sites, respectively. These observations are consistent with previous biochemical studies, in which both NOLC1 (also called Nopp140) and TCOF1 were able to undergo in vitro radiolabeling by [β^32^P]5PP-InsP_5_ (refs. ^15,16^). Both NOLC1 and TCOF1 are densely phosphorylated proteins, which presumably facilitates their pyrophosphorylation. Interestingly, the pyrophosphorylation sites on NOLC1 and TCOF1 exclusively localize to acidic regions, whereas phosphorylation sites are reported to be more evenly distributed across acidic and basic regions (Fig. 3a). The localization of pyrophosphorylation sites to acidic serine stretches was observed in previous studies on 5PP-InsP_5_-mediated pyrophosphorylation^16,18–20,31,32^. In all cases, the priming in vitro phosphorylation was catalyzed by acidophilic Ser/Thr kinases, especially casein kinase 2 (CK2). A global analysis of all endogenous pyrophosphorylation sites detected by MS in HEK293T and HCT116 lysates confirmed the central role for acidophilic Ser/Thr kinases—alignment of all sequences revealed a clear CK2 consensus sequence (Fig. 3b)^33^. However, some pyrophosphorylation sequences did not match the CK2 consensus sequence. When we removed all sites containing a Glu/Asp/Ser/Thr residue in position +3 from the alignment, a proline-directed kinase consensus sequence emerged (Fig. 3b)^34^, suggesting that this family of Ser/Thr kinases may also pre-phosphorylate residues prior to their pyrophosphorylation.

Of the 148 pyrophosphorylated sites identified in our study, only three occur on threonine residues, and the remainder are on serine (Fig. 3c). We analyzed the pyrophosphosites using the Scansite 4.0 tool to predict motifs that are likely to undergo phosphorylation by specific protein kinases (https://scansite4.mit.edu/#scanProtein)^34^. Consistent with the consensus sequences above, most of the sites were predicted to be phosphorylated by acidophilic Ser/Thr kinases; a smaller number were potential substrates for proline-directed Ser/Thr kinases; and a few sites may be substrates for both families of kinases (Fig. 3d). Approximately 7% of the mapped pyrophosphorylated residues were not predicted as substrates for either acidophilic or proline-directed Ser/Thr kinases, suggesting that other families of protein kinases may also prime residues for pyrophosphorylation. An additional feature common to pyrophosphorylation sites is that they lie within intrinsically disordered regions (IDRs)^17^. A disorder prediction analysis using IuPred2A (https://iupred2a.elte.hu/; Supplementary Table 3) showed that 91% of the pyrophosphosites identified in our study lie within a continuous stretch of 20 or more residues that have a disorder score ≥0.5, which is the cutoff for predicted disorder at that residue (Fig. 3e). This is in line with properties of phosphorylation sites in general, as IDRs are overrepresented in eukaryotic phosphoproteomic datasets^35,36^.

Gene Ontology analysis of pyrophosphorylation sites suggests a function for this modification in nuclear and nucleolar processes, as these two compartments were significantly overrepresented among pyrophosphorylated proteins (Fig. 3f and Supplementary Table 4). This localization preference is mirrored by the biological processes, in which RNA processing (specifically RNA splicing) emerged as a process putatively regulated by protein pyrophosphorylation of SRRM1, SRRM2, serine/arginine-rich splicing factor 2 (SRSF2), SRSF5, SRSF6, SRSF9, splicing factor 3B subunit 2(SF3B2), WW domain-binding protein 11 (WBP11) and transformer-2 protein homolog beta (TRA2B). Another biological process potentially regulated by pyrophosphorylated proteins is chromatin organization, which is modulated by epigenetic regulators, including high mobility group protein A1 (HMGA1) and the histone deacetylase HDAC2, among others. Previous studies showed that inositol pyrophosphates can influence epigenetic modifications that regulate chromatin remodelling in yeast and mammals^37^. Proteins NOLC1, nucleolar protein 58 (NOP58), nucleophosmin (NPM1), H/ACA ribonucleoprotein complex subunit DKC1 (DKC1) and U3 small nucleolar ribonucleoprotein protein MPP10 (MPHOSPH10) are involved in ribosome biogenesis, another process overrepresented among pyrophosphoproteins (Fig. 3f). A functional connection between PP-InsPs and ribosome biogenesis was previously made in *S. cerevisiae* using genetics, and a mechanistic hypothesis for this regulation involved pyrophosphorylation of the RNA polymerase I subunits A190, A43 and A34.5 (ref. ^32^).

### Pyrophosphoproteins in the nucleolar fibrillar center

**Fig. 4:**
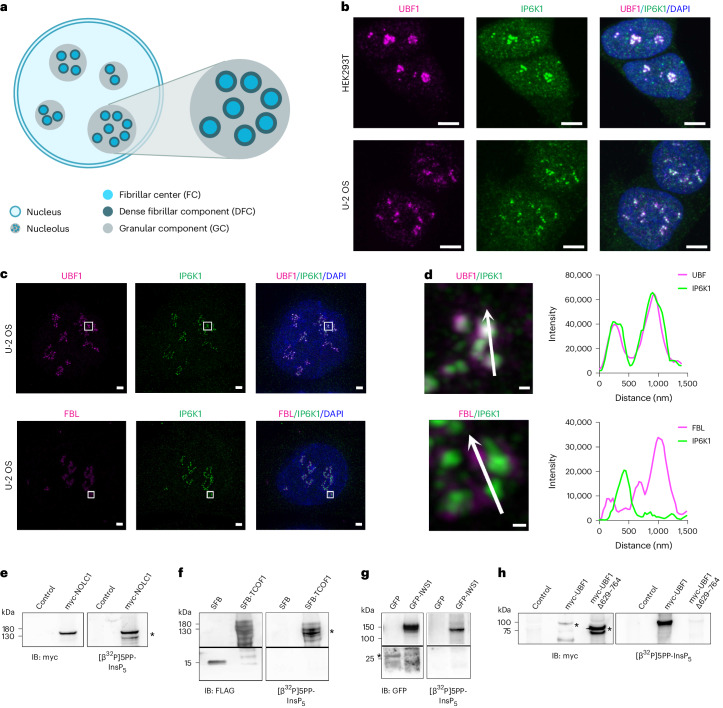
Nucleolar FC proteins undergo 5PP-InsP_5_-mediated pyrophosphorylation. **a**, Architecture of the mammalian nucleolus and its subcompartments. **b**, Confocal micrographs representative of three independent experiments showing co-localization of IP6K1 (green) with nucleolar FC marker UBF1 (magenta) in HEK293T and U-2 OS cells. Nuclei were stained with DAPI (blue); scale bars, 5 μm. **c**, SIM images representative of three independent experiments showing IP6K1 (green) co-localized with UBF1 or the nucleolar DFC marker FBL (magenta) in U-2 OS cells. Nuclei were stained with DAPI (blue); scale bars, 2 μm. **d**, Magnification of the boxed region (**c**); scale bars, 0.2 μm. Traces show fluorescence intensity profiles for IP6K1 (green) and UBF1 or FBL (magenta), measured along the indicated white arrow. **e**–**h**, In vitro pyrophosphorylation of human proteins by [β^32^P]5PP-InsP_5_. NOLC1, TCOF1, IWS1 and UBF1 (full-length and C-terminally deleted versions), with their indicated N-terminal tags, were expressed in HEK293T cells, isolated and prephosphorylated by CK2 before incubation with [β^32^P]5PP-InsP_5_. Representative images show autoradiography to detect pyrophosphorylation (right) and immunoblotting with respective tag-specific antibodies (left) (*n* = 4 (**e**), *n* = 5 (**f**), *n* = 4 (**g**) and *n* = 2 (**h**)). Negative controls were cells transfected with plasmids pCMV-myc (**e**,**h**), pCDNA-SFB (**f**) or EGFP-C1 (**g**). The asterisks in **e**–**h** indicate specific bands. IB, immunoblot. Source data

The nucleolus, which emerged as a major site for localization of pyrophosphorylated proteins, is a membrane-less organelle organized into three liquid–liquid phase-separated subcompartments: the fibrillar center (FC), the dense fibrillar component (DFC) and the granular component (GC) (Fig. 4a)^38^. rDNA repeats present in the FC are transcribed by RNA polymerase I at the FC/DFC boundary; the resulting pre-rRNAs are processed in the DFC and assembled into ribosomes in the GC^38^. Human IP6 kinase isoforms IP6K1 and IP6K2 are reported to be localized to the nucleolar FC region (Human Protein Atlas, https://www.proteinatlas.org/)^39^. We used immunofluorescence to confirm the co-localization of IP6K1 with the FC marker protein upstream binding factor 1 (UBF1)^40^ in HEK293T cells (Fig. 4b). The same pattern of localization was observed in the osteosarcoma cell line U-2 OS (Fig. 4b). Super-resolution microscopy revealed that IP6K1 is confined to the FC and does not co-localize with fibrillarin (FBL), which marks the DFC^41^ (Fig. 4c,d). Local synthesis of 5PP-InsP_5_ by IP6Ks in the FC would facilitate pyrophosphorylation of FC resident proteins. Indeed, the two most highly pyrophosphorylated proteins identified in our study, NOLC1 and TCOF1, are localized to the FC^42^. Four additional pyrophosphoproteins—NOP58; DKC1; SWI/SNF-related, matrix-associated, actin-dependent regulator of chromatin, subfamily A, member 4 (SMARCA4); and eukaryotic translation elongation factor 1 delta (EEF1D)—are also annotated to the FC^42^. UBF1, which co-localizes with IP6K1 in the FC (Fig. 4b–d), has a region of disorder at the C-terminus, which contains serine residues interspersed with glutamate and aspartate residues in a sequence that is reminiscent of the pyrophosphosites on TCOF1, NOLC1 and the nuclear protein IWS1 (Supplementary Fig. 4). UBF1 was, however, not identified in our pyrophosphoproteome MS screen, and so we resorted to radiolabeled [β^32^P]5PP-InsP_5_, which has been used as a classical tool to test for pyrophosphorylation of candidate proteins^21,43^. We confirmed that NOLC1, TCOF1 and IWS1 overexpressed and isolated from HEK293T cells can undergo in vitro pyrophosphorylation with [β^32^P]5PP-InsP_5_ after pre-phosphorylation by CK2 (Fig. 4e–g). Similarly, we observed robust pyrophosphorylation of CK2 pre-phosphorylated UBF1 upon exposure to [β^32^P]5PP-InsP_5_ (Fig. 4h). We predicted pyrophosphorylation sites on UBF1 by identifying regions of disorder that contain sites for pre-phosphorylation by acidophilic Ser/Thr kinases (Supplementary Fig. 4). Deletion of the C-terminal disordered region of UBF1, which possesses a large number of potential pyrophosphosites, abrogated in vitro pyrophosphorylation of the protein, confirming that UBF1 undergoes pyrophosphorylation at its C-terminus (Fig. 4h). Although UBF1 is a relatively abundant protein (Extended Data Fig. 4), the predicted pyrophosphosites in the C-terminus of UBF1 lie within either very short or very long tryptic fragments, both of which would evade detection by MS.

### PP-InsPs support pyrophosphorylation and rDNA transcription

**Fig. 5:**
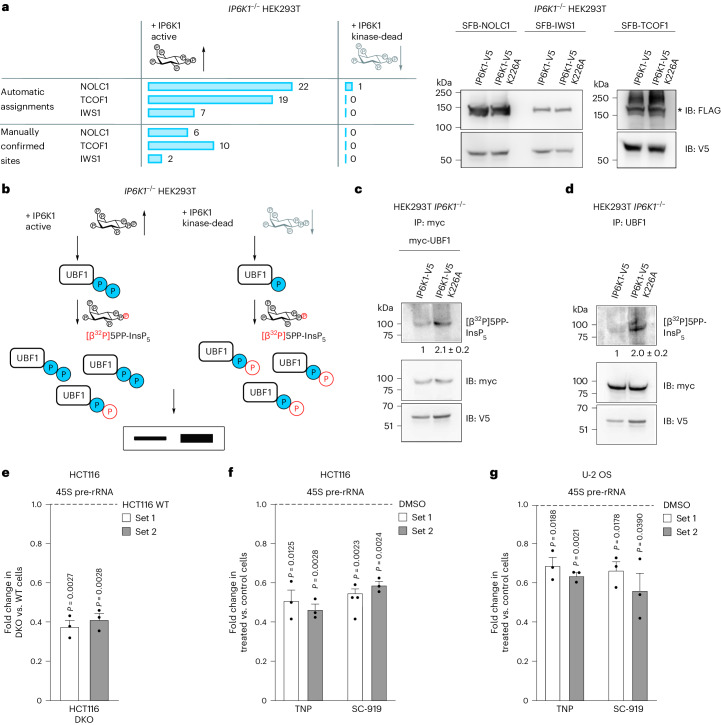
5PP-InsP_5_ drives cellular pyrophosphorylation and promotes rRNA synthesis. **a**, Comparative MS analysis of pyrophosphosites detected in SFB-tagged NOLC1, TCOF1 or IWS1 co-expressed with V5-tagged active or kinase-dead IP6K1 (left). Number of pyrophosphopeptides assigned by automatic or manual analysis. An additional analysis, including the number of EThcD triggers, can be found in Supplementary Fig. 5. Immunoblot (IB) shows that equal amounts of protein were subjected to MS (right). Asterisk indicates the specific band (*n* = 1). **b**, Back-pyrophosphorylation method to detect intracellular UBF1 pyrophosphorylation. **c**,**d**, Overexpressed myc-tagged UBF1 (**c**) or endogenous UBF1 (**d**) was immunoprecipitated from *IP6K1*^−/−^ HEK293T cells expressing either active or kinase-dead V5-tagged IP6K1 and incubated with [β^32^P]5PP-InsP_5_. Images show autoradiography to detect pyrophosphorylation (top) and immunoblotting with the indicated antibodies (bottom). IB with V5 antibody detects input levels of IP6K1. Numbers show mean fold change ± s.e.m. in the extent of UBF1 back-pyrophosphorylation in cells expressing kinase-dead compared to active IP6K1 (*n* = 3 (**c**) and *n* = 4 (**d**) independent experiments). **e**–**g**, RT–qPCR analysis to measure 45S pre-rRNA transcript levels using two different primer sets. Values indicate the fold change in transcript levels in *IP6K1*^−/−^*IP6K2*^−/−^ DKO cells compared to HCT116 WT cells (**e**) and TNP-treated or SC-919-treated HCT116 (**f**) or U-2 OS (**g**) cells compared to cells treated with the vehicle control (DMSO). Data are mean ± s.e.m. (*n* = 3 independent experiments). *P* values were determined using a two-tailed one-sample *t*-test. IP, immunoprecipitation. IB, immunoblotting. Source data

**Extended Data Fig. 5:**
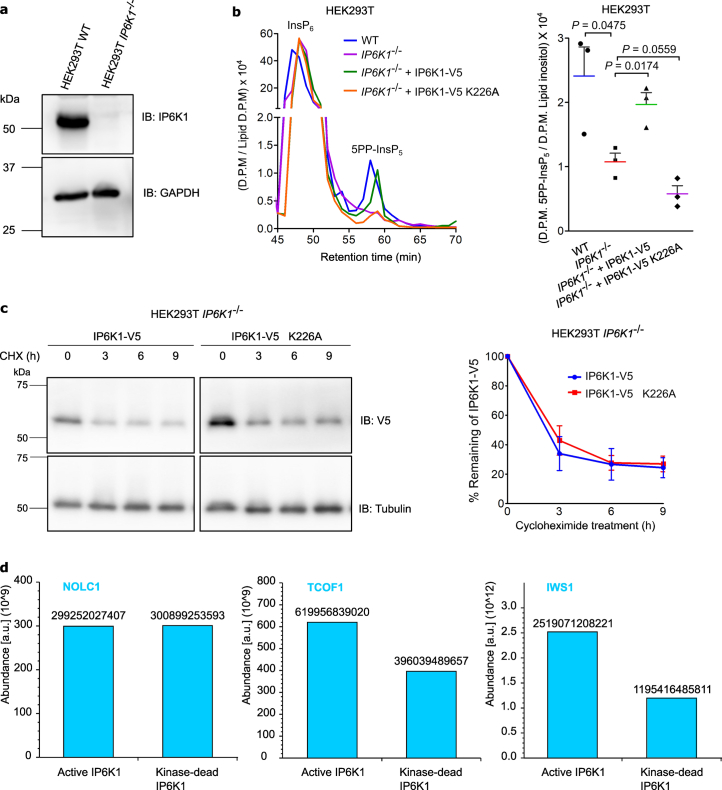
Controls for pulldown experiments. **a**) Representative immunoblots demonstrating the absence of IP6K1 in HEK293T *IP6K1*^−/−^ knockout cell line (n = 4). **b**) Left: representative HPLC profiles of [^3^H]-inositol labeled HEK293T wild type cells, *IP6K1*^−/−^ knockout cells, and *IP6K1*^−/−^ knockout cells expressing V5-epitope tagged active or kinase-dead IP6K1. Soluble inositol phosphate counts were normalized to the total lipid inositol count for each sample. Peaks corresponding to InsP_6_ and 5PP-InsP_5_ are indicated. Right: Level of 5PP-InsP_5_ normalized to the total lipid inositol in the four cell lines from a (mean ± SEM, n = 3 independent experiments) analyzed using a two-tailed unpaired Student’s t test. **c**) Protein stability of active and kinase-dead IP6K1 expressed in *IP6K1*^−/−^ HEK293T cells subjected to treatment with cycloheximide (100 µg/mL) to block protein synthesis for the indicated time. α-tubulin was used as a loading control. Graphs show the levels of IP6K1 at each time point, normalized to the untreated sample (mean ± SEM, n = 5 independent experiments). Loss of kinase activity did not alter the stability of IP6K1. **d**) Abundance of target proteins in pulldown samples. Comparison between samples from a background with high (IP6K1-kinase active, KA) or low (IP6K1-kinase dead, KD) PP-InsP levels. Source data

**Extended Data Fig. 6:**
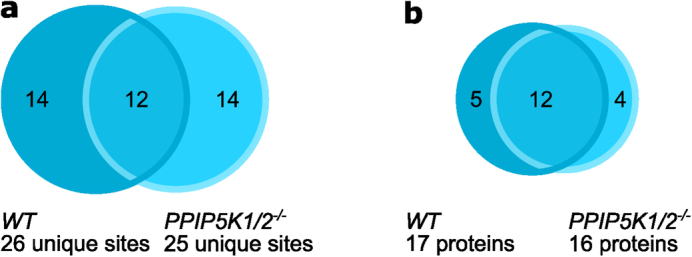
Pyrophosphoproteomics on HCT116 *PPIP5K1/2*^−/−^ cells. **a**) Overlap of manually validated pyrophosphorylation sites in wild type vs knockout cells. **b**) Overlap of modified proteins.

Given the complementarity of the radiolabeling and MS methods, we used both approaches to affirm that 5PP-InsP_5_ drives intracellular protein pyrophosphorylation. For this, we relied on *IP6K1*^−/−^ HEK293T cells expressing either active or kinase-dead IP6K1, which display a three-fold difference in the levels of 5PP-InsP_5_ (Extended Data Fig. 5a–c). TCOF1, NOLC1 or IWS1 expressed in these cells (Fig. 5a)^44^ were purified, treated with λ-phosphatase and resolved by SDS-PAGE, and the excised gel pieces were digested with trypsin and measured by neutral-loss-triggered EThcD MS as described above. Equal loading between conditions was confirmed by analyzing absolute abundance of target proteins using the Precursor Ions Quantifer node in Proteome Discoverer (Extended Data Fig. 5d). Many automatic pyrophosphorylation site assignments in the samples were obtained from cells expressing active IP6K1, several of which could be confirmed to correspond to pyrophosphorylated peptides for all three substrates (Fig. 5a and Supplementary Table 5). By contrast, we could not confirm pyrophosphorylation on a single peptide sequence triggered by the characteristic loss of the pyrophosphoryl moiety from the cells expressing kinase-dead IP6K1. These results illustrate the strong dependence of endogenous pyrophosphorylation on cellular PP-InsP levels and point to 5PP-InsP_5_ as the predominant phosphoryl donor. To interrogate the involvement of 1,5(PP)_2_-InsP_4_ in protein pyrophosphorylation_,_ we performed pyrophosphoproteomics on a lysate from *PPIP5K*^−/−^ HCT116 cells, which cannot produce 1,5(PP)_2_-InsP_4_ (ref. ^45^). Similar levels of pyrophosphorylation were observed in the *PPIP5K*^−/−^ cells compared to the wild-type (WT) cells, suggesting that 1,5(PP)_2_-InsP_4_ is not a major mediator of protein pyrophosphorylation in this cell line (Extended Data Fig. 6 and Supplementary Table 6). However, caution must be taken when interpreting these results, as *PPIP5K*^−/−^ cell lines produce increased levels of 5PP-InsP_5_ (refs. ^46,47^), which may compensate for a loss of 1,5(PP)_2_-InsP_4._

As pyrophosphosites on UBF1 evade detection by MS, we resorted to the ‘back-pyrophosphorylation’ method to examine intracellular UBF1 pyrophosphorylation^21^ (Fig. 5b). Overexpressed or endogenous UBF1 was isolated from *IP6K1*^−/−^ HEK293T cells expressing either active or kinase-dead IP6K1 and incubated with radiolabeled [β^32^P]5PP-InsP_5_. UBF1 isolated from cells expressing kinase-dead IP6K1 showed a two-fold higher pyrophosphorylation signal on the autoradiogram compared to UBF1 from cells with active IP6K1, reflecting higher levels of intracellular pyrophosphorylation on the latter form of UBF1 (Fig. 5c,d). The increase in cellular pyrophosphorylation on TCOF1, NOLC1, IWS1 and UBF1 in the presence of active IP6K1 is consistent with intracellular pyrophosphorylation mediated by 5PP-InsP_5_.

TCOF1, NOLC1 and UBF1 are known regulators of RNA polymerase I–mediated rDNA transcription^38,39,48,49^. Reduced intracellular 5PP-InsP_5_, which, in turn, will lower pyrophosphorylation levels of these proteins, is therefore likely to have an impact on rRNA synthesis. To examine this possibility, we used two different cellular models known to be depleted for 5PP-InsP_5_: (1) HCT116 cells lacking IP6K1 and IP6K2 (ref. ^50^) and (2) cells treated with the InsP_6_ kinase inhibitors TNP or SC-919 (refs. ^51,52^). Quantitative RT–PCR analysis was used to assess the levels of the 45S pre-rRNA transcript, which is subsequently processed to yield mature rRNA for incorporation into ribosomes. We observed a more than two-fold decrease in the levels of 45S pre-rRNA in *IP6K1*^−/−^*IP6K2*^−/−^ double knockout (DKO) cells compared to WT HCT116 cells (Fig. 5e). Treatment with IP6K inhibitors recapitulated these findings and similarly suppressed pre-rRNA synthesis in HCT116 and U-2 OS cells, compared to cells treated with the vehicle control (Fig. 5f,g). These observations point to an important role for 5PP-InsP_5_ in the maintenance of rDNA transcription, likely via pyrophosphorylation of key nucleolar proteins.

## Discussion

In this work, we developed a tailored pyrophosphoproteomics workflow to detect and assign protein pyrophosphorylation in two human cell lines (148 manually validated sites across 71 proteins), providing, to our knowledge, the first direct evidence of endogenous protein pyrophosphorylation. Over time, efforts to characterize non-canonical phosphorylation with phosphoproteomic methods have advanced, as sample handling, enrichment and MS techniques have been adapted to the properties of the modification. For example, pHis proteomics has evolved by leveraging antibody enrichment and, later, strong anion exchange, culminating in a dedicated pHis database, HisPhosSite, containing more than 270 putative human sites^53^. Despite these advances, site localization and, therefore, distinction from canonical phosphorylation has remained a major technical challenge. As site localization probabilities are tightened in assignment workflows, unambiguous assignments are markedly reduced^8^. The pHis immonium ion was recently used as additional evidence of correct pHis assignment, but only 0.5% of putative pHis-containing peptides in a human dataset exhibited this ion, suggesting significant overassignment by current automated methods^14^.

In the case of pyrophosphorylation, with our tailored sample preparation workflow and manual assignment strategy, we could achieve unambiguous pyrophosphosite assignment. The proteomic datasets indicated that pyrophosphorylation often occurred in acidic stretches known to be multiply phosphorylated, meaning that mixtures of isobaric bisphosphopeptide isomers and pyrophosphopeptides may be co-fragmented during EThcD, increasing the risk of false assignment. To exclude this possibility, both the CID and EThcD fragmentation patterns were assessed, and spectra containing monophosphorylated fragments were excluded from the analysis. Although this manual workflow is more laborious than automated methods, the assigned pyrophosphorylation sites are unambiguous and can be directly used to inform further biological investigation. We expect that it is possible to integrate the data analysis into software-based assignment, avoiding manual assessment of sites in the future. Even with the current automated assignment alone, an indicative number of pyrophosphorylation sites are identified, sufficient for preliminary investigations.

The dependence of pyrophosphorylation on PP-InsPs was demonstrated based on the lack of characteristic neutral loss triggers and assigned pyrophosphosites originating from NOLC1, TCOF1 and IWS1 proteins expressed in a PP-InsP-depleted (IP6K1-kinase dead) cell line. All three proteins were also able to accept the radiolabeled phosphoryl group from [β^32^P]5PP-InsP_5_, demonstrating that the pyrophosphorylation of these proteins is consistent with the non-enzymatic model of protein pyrophosphorylation.

In the future, a quantitative MS approach likely involving tandem mass tag (TMT) labeling or stable isotope labeling by amino acids in cell culture (SILAC) will be needed to quantify levels of protein pyrophosphorylation in different cellular backgrounds or treatment conditions. This approach would also be required to investigate site occupancy; as peptides are deliberately dephosphorylated during sample preparation, comparing amounts of pyrophosphopeptides and their phosphorylated or unmodified precursors in a single analysis is not possible. Further optimization to reduce the quantity of material required per analysis and to improve the level of overlap between biological replicates would make the method more accessible. Both issues likely relate to inefficiencies in the λ-phosphatase treatment and enrichment steps, causing non-pyrophosphorylated material to remain after sample preparation and stochastically mask pyrophosphopeptide ions during MS analysis. Implementation of online Fe-IMAC chromatography^54^ might allow for improved selectivity, reducing material requirements and increasing reproducibility. Optimizing the λ-phosphatase reaction would further reduce the selectivity requirement of the enrichment step. Additionally, the lack of Lys/Arg residues for tryptic cleavage in acidic polyserine stretches may prevent detection of certain pyrophosphopeptides with our approach. Pyrophosphorylation of residues adjacent to the cleavage site may also prevent protein digestion. In the future, these possibilities could be addressed by integrating additional proteases, such as GluC, into the digestion step alongside trypsin^55^.

NOLC1 and TCOF1 were the two most heavily pyrophosphorylated proteins identified in this study. Along with UBF1, here characterized as a novel in vitro pyrophosphorylation substrate, NOLC1 and TCOF1 are known to upregulate rRNA synthesis via RNA polymerase I binding^56–59^. CK2-dependent phosphorylation at the C-terminal region of UBF1 promotes its interaction with SL1 to form a stable RNA polymerase I pre-initiation complex^59,60^. As most of the predicted UBF1 pyrophosphorylation sites lie within its C-terminus (Supplementary Fig. 4), pyrophosphorylation on UBF1 after CK2 priming may conceivably regulate rRNA synthesis. Depletion of 5PP-InsP_5_ leads to decreased rDNA transcription (Fig. 5e–g), consistent with regulation via polymerase I. We previously showed that, in budding yeast *S. cerevisiae*, the absence of 5PP-InsP_5_ leads to severely reduced rRNA synthesis, correlating with pyrophosphorylation of RNA polymerase I subunits^32^. A recent study showed that elevation of intracellular 5PP-InsP_5_ in *PPIP5K*^−/−^ HCT116 cells does not alter the steady state levels of rRNA, but this work did not examine pre-rRNA transcript levels that reflect rDNA transcription by polymerase I (ref. ^61^). 5PP-InsP_5_ is thought to be a ‘metabolic messenger’ as its synthesis by IP6Ks is uniquely sensitive to ATP availability^62,63^. If the interaction between NOLC1/TCOF1/UBF1 and RNA polymerase I were indeed dependent on 5PP-InsP_5_-mediated pyrophosphorylation, this would represent a straightforward cellular energy sensing mechanism to control rDNA transcription.

The localization of pyrophosphoproteins to the nucleolus was striking and raises the question of whether pyrophosphorylation plays a general regulatory role in this biomolecular condensate. It was recently demonstrated that phosphorylation of specific sites influenced partitioning of NPM1 (pSer125) and heterogeneous nuclear ribonucleoprotein A1 (HNRNPA1) (pSer6) to the nucleolus^64^. Interestingly, both proteins were found to be pyrophosphorylated at these specific positions in our MS data. It is, therefore, valuable to develop tools that can accurately represent or mimic pyrophosphorylation at the protein level. Such tools would enable researchers to investigate the influence of pyrophosphorylation on protein partitioning into membrane-less condensates.

Overall, protein pyrophosphorylation has emerged as an abundant, non-canonical phosphorylation. The ability to detect this modification within complex samples using MS and to definitively assign the modification sites now opens the door to investigate the functional role of protein pyrophosphorylation at the biochemical and cellular level. Although the installation of pyrophosphorylation appears to be non-enzymatic in biochemical assays, the question remains as to whether the presence of cell lysates or other co-factors can accelerate protein pyrophosphorylation. How the features of the PP-InsP phosphoryl donor influence the degree and the specificity of pyrophosphorylation has not been addressed to date. After installation, pyrophosphorylation could be detected by specific reader domains, and characterization of the pyrophosphoproteome will facilitate the identification of such readers. Finally, the removal of pyrophosphorylation sites will need to be explored. Are there dedicated protein pyrophosphatases that convert pyrophosphoserine back to phosphoserine or serine? Answers to these questions will provide fundamental insight into the regulation of protein pyrophosphorylation and its interplay with signaling pathways controlled by canonical protein phosphorylation.

## Methods

### Pyrophosphoproteomics sample preparation workflow

#### Cell lysis and digestion

HEK293T cells (American Type Culture Collection, CRL-3216) were cultured by seeding 2 × 10^6^ cells into eight 15-cm culture plates in DMEM (5% FBS, 1 mM L-glutamine, 50 U ml^−1^ penicillin, 100 µg ml^−1^ streptomycin). Plates were cultured for 3 d, with medium exchange on day 3 and harvest on day 4 (80–90% confluency). To lyse, plates were washed twice with 5 ml of 0.9% NaCl, and then 2 ml of lysis buffer (8 M urea, 75 mM NaCl, 50 mM Tris (pH 8.2), 1 mM NaF, 1 mM β-glycerophosphate, 1 mM sodium orthovanadate, 10 mM sodium pyrophosphate, 1 mM PMSF, one cOmplete EDTA-free protease inhibitor tablet (Roche) per 10 ml)^24^ was added to seven plates. Plates were incubated at 4 °C for 10–15 min. The remaining plate was trypsinized, and cells were counted using a Bio-Rad cell counter (average cell number in HEK293T triplicate: 6.6 × 10^6^ cells per milliliter).

Lysed plates were scraped using a spatula, and the combined lysate was transferred into a 50-ml Falcon tube. The lysate was subjected to sonication at 4 °C (on ice, 50% output, 0.5 cycle rate, 5× 30 s, 30-s rest between pulses). The lysate was centrifuged (3,200*g*, 10 min, 4 °C); aggregated insoluble material on the surface was removed; and the supernatant was decanted from the cell pellet and retained. Supernatant protein concentration was determined by BCA assay (commercial kit by Thermo Fisher Scientific, average 112 mg).

To reduce and alkylate lysate proteins, DTT in Milli-Q water (5 mM concentration in sample) was added, and the sample was incubated at 37 °C for 1 h. After incubation, the sample was cooled to room temperature. Iodoacetamide in Milli-Q water (14 mM concentration in sample) was added, and the sample was incubated at room temperature in the dark for 30 min. Remaining iodoacetamide was quenched with a second aliquot of DTT (10 mM final concentration in sample), and the sample was incubated for 15 min at room temperature in the dark.

To digest, sequencing-grade modified trypsin (Promega, V5111) was used. On ice, the lysate was diluted approximately 1:5 with 25 mM Tris (pH 8.0). CaCl_2_ (1 mM concentration in sample) was added. Trypsin was added at a ratio of approximately 1:50 protease to protein. The sample was incubated for 16 h at 37 °C with 500-r.p.m. agitation.

#### Lysate desalting

Neat TFA (approximately 0.4% of sample volume) was added (approximately pH 2), and the sample was centrifuged for 10 min at 3,200*g*. The supernatant of the centrifuged protein solution was desalted using four SepPak tC18 3-cc 500-mg cartridges (Waters, WAT043425; loading of approximately 20 mg per cartridge). Air pressure was used to speed up washing steps but was not used during the loading or elution steps. To prepare, columns were washed with MeCN (9 ml) and then with 3 ml of 50% MeCN in water with 0.5% AcOH. The columns were equilibrated with 0.1% TFA in water (9 ml) and then each loaded with the digested peptide samples in 0.4% TFA in water. The columns were washed with 0.1% TFA in water (9 ml) to desalt the peptides. The counter-ion was exchanged by a final wash with 0.5% AcOH in water (1 ml). Peptides were eluted with 6 ml of 50% MeCN and 0.5% AcOH in water. The eluent was lyophilized overnight to isolate tryptic digest as a white solid (average yield 84 mg)

#### λ-phosphatase treatment

Lyophilized tryptic digest (50 mg) was dissolved in 10 ml of phosphatase reaction buffer (50 mM HEPES, 100 mM NaCl, 1 mM MnCl_2_, 2 mM DTT, 0.01% Brij 35). The solution was then treated with 50,000 units of λ-phosphatase and incubated at 37 °C with 300-r.p.m. agitation for 5 h. Neat TFA was added (approximately 0.4% of sample volume, to pH 2), and the sample was centrifuged (10 min, 3,200*g*) to remove any cellular debris. The samples were then desalted as described in the previous step, except that only three SepPak cartridges were used. The eluent was again lyophilized to isolate the tryptic digest as a white solid (average yield 45 mg).

#### SIMAC enrichment

A High Select Fe-NTA Phosphopeptide Enrichment Kit from Thermo Fisher Scientific was used to enrich the pyrophosphopeptides using a protocol adapted from the manufacturer’s instructions to facilitate SIMAC^26^.

Tryptic digest (40 mg) treated with λ-phosphatase from the previous step was dissolved in 800 µl of SIMAC loading buffer (0.1% TFA, 50% MeCN). Four columns were washed twice with 200 µl of SIMAC loading buffer and then closed with a plug supplied with the kit and loaded with 200 µl of the digest solution (10 mg per column). The columns were incubated for 30 min at room temperature. Every 10 min, the columns were gently tapped for 10 s to resuspend the Fe-NTA resin in the peptide solution.

After this binding step, columns were washed three times with 200 µl of SIMAC loading buffer, once with 200 µl of Milli-Q water, twice with 100 µl of SIMAC buffer A (1% TFA, 20% ACN, to selectively elute monophosphopeptides) and once again with 200 µl of Milli-Q water.

Elution was performed twice with 100 µl of SIMAC buffer B (0.5% NH_4_OH in water) into clean 2-ml Protein LoBind Eppendorf tubes. The eluent from four columns was then combined into a single 2-ml Protein LoBind Eppendorf tube. This yielded an 800-µl peptide solution that was directly frozen using liquid nitrogen and lyophilized overnight.

#### Fractionation

After enrichment, the material was dissolved in 100 µl of high-performance liquid chromatography (HPLC) buffer A, spun at 21,000*g* for 10 min to pellet any insoluble components and transferred to an HPLC vial for fractionation. Separation was achieved using a decreasing pH gradient to exploit pKa differences among acidic amino acid side chains, phosphoryl groups and pyrophosphoryl groups, with lower pKa peptides expected to retain longer. Additionally, a decreasing acetonitrile gradient was employed with the aim to rapidly elute peptides with low negative charge in the early fractions of the separation. Fractionation was performed on an Agilent 1260 Infinity HPLC, equipped with an IonPac AS24 2-mm analytical ultra-hydrophilic SAX column (Thermo Fisher Scientific) and the corresponding IonPac AG24 guard column. HPLC buffers were prepared freshly.

HPLC buffer A: 20% MeCN in distilled water, 1% formic acid, pH 9.0 set with NH_4_OH. Buffer B: 1% MeCN, 1% NH_4_OH, pH 2.8 set with formic acid. Flow rate: 0.2 ml min^−1^. Gradient: 0–5 min, 100% A; 5–50 min, gradient increase to 100% B, 50–60 min, 100% B. Sample injection volume was 100 µl. Fractions were collected every 5 min, 12 1-ml fractions in total.

Fractions were transferred to 2-ml Protein LoBind Eppendorf tubes and lyophilized overnight. The resulting solids were redissolved in 200 µl of 20% MeCN and transferred to glass LC–MS vials and then dried using a vacuum centrifuge for 2 h at room temperature. Samples were stored at −20 °C until LC–MS analysis.

### LC–MS

Fractions were re-suspended with 50 mM citric acid in 3% acetonitrile solution before submitting to the LC–MS analysis. At this concentration, citric acid was tolerated by the LC–MS system and 50 mM was never exceeded. Each biological replicate was injected in two technical replicates, and the identified sites were combined. Sample separation was achieved by reverse-phase HPLC on a Thermo Fisher Scientific Dionex UltiMate 3000 system coupled online to an Orbitrap Fusion mass spectrometer (Thermo Fisher Scientific), operated with the Xcalibur software package (Thermo Fisher Scientific) version 4.4.16.14. For sample loading, a PepMap C18 trap column (Thermo Fisher Scientific) of 0.075 mm ID × 50 mm length, 3-μm particle size and 100-Å pore size was used. Reversed-phase separation was performed using a 50-cm analytical column (in-house packed with Poroshell 120 EC-C18, 2.7 µm, Agilent Technologies) with mobile phase A containing 0.1% formic acid in water and mobile phase B containing 0.1% formic acid in acetonitrile. The gradient started with 4% mobile phase B reaching 80% mobile phase B in 101 min, with total run time of 120 min, including column wash and equilibration.

MS1 scans were acquired in the Orbitrap with a mass resolution of 120,000. MS1 scan range was set to 380–1,400 *m*/*z*, with standard AGC target of 4 × 10^5^ and maximum injection time of 50 ms. Precursor ions with charge states +2 to +4 were isolated with an isolation window of 1.6 *m*/*z* and dynamic exclusion of 15 s. Precursor ions were selected with precursor priority to the higher charge state. MS2 scans were acquired in the Orbitrap with AGC target of 1 × 10^4^ and maximum injection time of 100 ms. Precursor ions were fragmented using CID with a normalized collision energy of 25%. If neutral losses of 177.9432 Da from precursor ions were measured above a threshold of 15% of relative intensity in the CID scan, an additional spectrum of the same precursor ions was acquired using EThcD. EThcD spectra were measured in the Orbitrap with a resolution of 15,000 with AGC target of 1 × 10^5^, maximum injection time of 2 s and normalized collision energy of 30%. Cycle time was set to 3 s.

### Sample preparation and LC–MS for intensity-based absolute quantification calculation

HEK293T cells were grown and harvested, and proteins were reduced, alkylated and digested with trypsin as described above. Then, 150 µg of peptides was dissolved in 10 mM NH_4_OH and loaded onto a Gemini 3-µm C18 110-Å 100 × 1-mm high pH suitable column. Peptides were separated using an 85-min gradient with 10 mM NH_4_OH in ACN as the eluent. All 24 fractions were dried in the SpeedVac, resuspended in 1% ACN/0.05% TFA and subjected to LC–MS. Online separation of the samples was achieved by reverse-phase HPLC on a Thermo Fisher Scientific Dionex UltiMate 3000 system connected to a PepMap C18 trap column (Thermo Fisher Scientific) and an in-house packed C18 column for reverse-phase separation at 300 nl min^−1^ flow rate over a 120-min gradient. Samples were analyzed on an Orbitrap Fusion mass spectrometer with Instrument Control Software version 3.4. Data were acquired in DDA mode. MS1 scans were acquired in the Orbitrap with a mass resolution of 120,000. MS1 scan range was set to 375–1,500 *m*/*z*, 100% normalized AGC target, 50-ms maximum injection time and 40-s dynamic exclusion. MS2 scans were acquired in the ion trap in rapid mode. The normalized AGC target was set to 100%, 35-ms injection time and an isolation window of 1.6 *m*/*z*. Only precursors at charged states +2 to +4 were subjected to MS2. Peptides were fragmented using NCE 30%. Raw files were searched using MaxQuant (version 1.6.2.6) using the following parameters: MS1 mass tolerance, 10 ppm; MS2 mass tolerance, 0.5 Da; maximum number of missed cleavages, 2; minimum peptide length, 7; peptide mass, 500–4,600 Da; protein and PSM false discovery rate (FDR) 1%. Carbamidomethylation (+57.021 Da) on cysteines was used as a static modification. Oxidation of methionine (+15.995 Da) and acetylation of the protein N-terminus (+42.011 Da) were set as a variable modification. Data were searched against the human proteome retrieved from UniProt with intensity-based absolute quantification (iBAQ) calculation and match between runs activated. Data visualization was performed in R.

### Data analysis

Raw files were analyzed using Proteome Discoverer (Thermo Fisher Scientific) version 2.4.

EThcD spectra were selected by spectrum selector, accepting precursor masses of 350–5,000 Da.

Non-fragment filter was used as follows: Precursor ions were removed within a 1-Da window offset, and charge-reduced precursors and neutral losses were removed within a 0.5-Da window offset.

The SEQUEST HT node was used for peptide identification, searching against a full human proteome, digested by trypsin, with two missed cleavages allowed. Precursor mass tolerance was 10 ppm, and fragment mass tolerance was 0.02 Da. Carbamidomethylation (C) was set as a static modification, and phosphorylation (S,T,Y), pyrophosphorylation (S,T) and oxidation (M) were searched as dynamic modifications.

PSMs were filtered using the fixed value PSM validator, with a maximum accepted delta Cn of 0.5.

ptmRS was used for automated PTM assignment. PhosphoRS mode was set to false to detect phosphorylation and pyrophosphorylation in parallel.

### Validation of individual workflow steps

A set of five synthetic pyrophosphopeptides with a variety of sequence characteristics was produced as previously published^66^. In brief, a phosphorimidazolide reagent was used to install a benzyl-protected β-phosphate onto a phosphoserine or phosphothreonine residue in a peptide, followed by Pd-catalyzed hydrogenolysis of the protecting group and purification by C8 reverse-phase HPLC. These standard peptides were used to validate individual steps of the sample preparation workflow. The sequences were the following:

- ppT-1: DAVTY-ppT-EHAK
- ppS-2: SQYHVDG-ppS-LEK
- ppS-3: LD-ppS-EEDSAWPTNEK
- ppS-4: NEEDEGH-ppS-NSSPR
- ppS-5: AQWTQE-ppS-FQSNNTR

#### λ-phosphatase treatment

Five synthetic pyrophosphopeptides (20 pmol each) and 0.5 µg of HCT116 tryptic digest were spiked into λ-phosphatase buffer (50 mM HEPES, 100 mM NaCl, 1 mM MnCl_2_, 2 mM DTT, 0.01% Brij 35). Total peptide amount was approximately 0.7 µg. λ-phosphatase was added (1 U µg^−1^ as in the proteomics workflow), and samples were incubated at 37 °C and 300 r.p.m. for 5 h in a heater shaker.

Four replicates containing PP peptides, four negative control samples without enzyme, four replicates containing the corresponding monophosphopeptides and four control samples with monophosphopeptides, but without enzyme, were treated in this way.

All samples were desalted using C18 stage tips and subjected to LC–MS/MS analysis as described above.

Extracted total ion counts (TICs) of +2 precursor masses of the synthetic phosphopeptides and pyrophosphopeptides were integrated on MS1 level and normalized against total background to achieve a relative quantification of peptide abundance in samples versus controls.

#### SIMAC enrichment

Four replicates containing 5 mg of a λ-phosphatase-treated tryptic digest from HCT116 cells and five synthetic pyrophosphopeptides (20 pmol each) and four controls containing only the HCT116 digest were dissolved in 200 µl of SIMAC loading buffer per sample (0.1% TFA, 50% MeCN) and subjected to SIMAC enrichment as in the pyrophosphoproteomics workflow described above. After enrichment, 20 pmol of each synthetic peptide was added to each control sample (positive control). Eluates were lyophilized, cleaned using C18 stage tips and dried by vacuum centrifugation. Peptides were redissolved in 6 µl of injection buffer (50 mM sodium citrate, 3 % MeCN) and subjected to LC–MS analysis as described above.

Extracted TICs of +2 precursor masses of the synthetic phosphopeptides and pyrophosphopeptides were integrated on MS1 level and normalized against total background to achieve a relative quantification of peptide abundance in samples versus controls.

#### Use of citrate resuspension buffer

Five synthetic pyrophosphopeptides (2 µl of peptide mix, 20 pmol each) and 0.5 µg of HCT116 tryptic digest (1 µl) were mixed with either 3 µl of water or 3 µl of citrate buffer (100 mM sodium citrate, 6% MeCN) and subjected to LC–MS analysis as described above.

Ion chromatograms of the most abundant free and iron-bound species [M+Fe(III)-H]^2+^ and [M + 2H]^2+^ were extracted, integrated and normalized against total background to achieve a relative quantification of free versus iron-bound pyrophosphopeptides. The same experiment was performed with the corresponding monophosphopeptides.

### Expression constructs

The plasmids employed for expression in mammalian cells are as follows: N-terminally SFB (S-protein/FLAG/SBP)-tagged human TCOF1 (GenBank ID NM_000356.4; gift from Maddika Subba Reddy, Centre for DNA Fingerprinting and Diagnostics); human UBF1 cDNA (GenBank ID NM_014233.4; gift from Solomon Snyder, Johns Hopkins School of Medicine) subcloned into pCMV-Myc-N plasmid for expression with an N-terminal myc tag; UBF1 Δ629-764 cloned into N-terminal myc-tagged destination vector using the Gateway cloning strategy (Thermo Fisher Scientific); human IWS1 (GenBank ID NM_017969.3) amplified using cDNA from HepG2 cells and cloned into N-terminal GFP or N-terminal SFB-tagged destination vectors; and N-terminally myc-tagged NOLC1 cDNA (GenBank ID NM_001284388.2) plasmid obtained from Sino Biological (HG16317-NM). The generation of catalytically active and inactive versions of C-terminally V5-tagged human IP6K1 (GenBank ID NM_001242829.2) was described previously^44^.

### Cell lines and transfection

Cell lines were grown in DMEM supplemented with 10% FBS, 1 mM L-glutamine, 100 U ml^−1^ penicillin and 100 µg ml^−1^ streptomycin in a humidified incubator with 5% CO_2_. Cell culture reagents were from Thermo Fisher Scientific. Cells were transfected using polyethylenimine (PEI) (Polysciences) at a ratio of 1:3 (DNA:PEI) and harvested 48 h after transfection for further analyses. WT and *IP6K1*^−/−^*IP6K2*^−/−^ DKO HCT116 cells were a gift from Adolfo Saiardi (University College London)^50^. *IP6K1*^−/−^ HEK293T cell line was generated using the CRISPR–Cas9 strategy. An sgRNA sequence targeting exon 5 of IP6K1 (Supplementary Table 7) was designed using the Benchling tool (https://www.benchling.com) and cloned into pU6-2A-GFP-2A-Puro plasmid (a gift from P. Chandra Shekar, Centre for Cellular & Molecular Biology), which co-expresses Cas9. The plasmid was transfected into HEK293T cells, and, 48 h after transfection, cells were selected using 2 μg ml^−1^ puromycin for 3 d. Serial dilution was performed to isolate single-cell-derived colonies, which were screened for frameshift mutations by genotyping using a 3500xL Genetic Analyzer (Applied Biosystems). Primers used for genotyping are listed in Supplementary Table 7. IP6K1 knockout was confirmed by immunoblot analysis

### Analysis of cellular inositol pyrophosphates

HEK293T WT and *IP6K1*^−/−^ cells expressing either active or kinase-dead (K266A) IP6K1 were seeded in 60-mm dishes and labeled with [^3^H]-inositol as described previously^67^. Upon attaining 30% confluence, cells were transferred to inositol-free DMEM (MP Biomedicals, D9802-06.25) containing 10% dialyzed FBS and 30 µCi myo-2-[^3^H] inositol (American Radiolabeled Chemicals, ART 0116B) for 2.5 d. The media were removed, and fresh media containing myo-2-[^3^H] inositol (30 µCi) were added for another 2.5 d, when plasmids expressing IP6K1-V5 or IP6K1-V5 K226A were transfected into *IP6K1*^−/−^ cells. Upon achieving isotopic labeling, cells were collected in chilled PBS. Soluble inositol phosphates were extracted by the addition of 350 µl of extraction buffer (0.6 M HClO_4_, 2 mM EDTA, 0.2 mg ml^−1^ phytic acid) for 15–20 min, followed by centrifugation at 21,000*g* for 10 min. The supernatant containing soluble inositol phosphates was collected, and lipid inositides in the pellet were extracted with 1 ml of lipid extraction buffer (0.1 N NaOH, 0.1% Triton X-100) at room temperature and counted in a liquid scintillation counter (Tri-Carb 2910 TR, PerkinElmer). The soluble inositol phosphate extract was mixed with approximately 120 µl of neutralization solution (1 M K_2_CO_3_, 5 mM EDTA). Tubes were left open on ice for 1 h, followed by centrifugation at 21,000*g* for 10 min at 4 °C. The extracted inositol phosphates were resolved by HPLC (515 or 5125 HPLC pumps, Waters) on a PartiSphere SAX column (4.6 mm × 125 mm, HiChrome) using a gradient of buffer A (1 mM EDTA) and buffer B (1 mM EDTA and 1.3 M (NH_4_)_2_HPO_4_ (pH 3.8)) as follows: 0–5 min, 0% B; 5–10 min, 0–20% B; 10–70 min, 20–100% B; 70–80 min, 100% B. Then, 1-ml fractions containing soluble inositol phosphates were mixed with 3 ml of scintillation cocktail (Ultima-Flo AP) and counted. The soluble inositol phosphate in each fraction was normalized to total lipid inositide in the sample.

### Immunofluorescence, protein pulldown and immunoblotting

The antibodies used in this study are listed in Supplementary Table 8. For immunofluorescence, cells seeded on glass coverslips were fixed with 4% paraformaldehyde for 15 min and permeabilized using PBS containing 0.15% Triton X-100 (PBST) for 15 min at room temperature. Cells were incubated with blocking solution (5% BSA in PBST) for 1 h at room temperature and then with primary antibodies diluted in the blocking solution overnight at 4 °C. Cells were washed thee times with PBST and incubated with fluorophore-conjugated secondary antibodies for 1 h at room temperature. After incubation, the cells were washed with PBST and mounted on glass slides using mounting medium with DAPI (Vector Laboratories). Images in Fig. 4b were captured on a Leica TCS SP8 confocal microscope equipped with 405-nm, 488-nm, 514-nm, 561-nm and 633-nm lasers using a ×63 1.4 NA oil immersion objective. Images in Fig. 4c,d were captured using the Elyra 7 structured illumination microscopy (SIM) module of the Zeiss LSM 980 confocal microscope, equipped with 405-nm, 488-nm, 561-nm and 642-nm lasers using a ×63 1.4 NA oil immersion objective. All immunofluorescence images are in z-stacks and are shown as maximum intensity projections using LAS X (Fig. 4b) or ZEN (Fig. 4c,d) software.

For protein pulldown, cells were harvested 48 h after transfection and lysed for 1 h at 4 °C in 1% lysis buffer (50 mM HEPES, pH 7.4, 150 mM NaCl, 1% Nonidet P-40, 1 mM EDTA, protease and phosphatase inhibitor cocktail) followed by sonication for 10 s at 30% amplitude (SONICS, VCX 750). To the lysate, the specific antibody was added and incubated overnight at 4 °C. The antibody-bound protein was pulled down using Protein A or Protein G Sepharose beads (GE Healthcare) for 1 h. The beads were washed with lysis buffer and used for [β^32^P]5PP-InsP_5_-mediated pyrophosphorylation. For SFB-tagged proteins, streptavidin sepharose beads (GE Healthcare) were added to the cell lysate and incubated at 4 °C before washing and pyrophosphorylation with [β^32^P]5PP-InsP_5_. For MS analysis of overexpressed proteins (Fig. 5a), 4% of cell lysate was used for immunoblotting, and 90% was pulled down on streptavidin sepharose beads. The beads were washed with lysis buffer and boiled in 1× Laemmli buffer, and proteins were resolved by SDS-PAGE. The bands were visualized with 0.2% Coomassie brilliant blue R-250, excised, digested in-gel with trypsin and subjected to neutral-loss-triggered EThcD MS as described above. For immunoblotting, proteins were transferred to a PVDF membrane and detected using standard western blotting techniques with protein-specific or tag-specific antibodies. To monitor protein stability, *IP6K1*^−/−^ HEK293T cells were transfected to express either active IP6K1-V5 or kinase-dead IP6K1-V5 (K226A), and, 24–30 h after transfection, cells were treated with cycloheximide (100 µg ml^−1^) for the indicated time. Cells were lysed for 1 h at 4 °C in lysis buffer (50 mM HEPES, pH 7.4, 100 mM NaCl, 0.5% Nonidet P-40, 1 mM EDTA, containing protease and phosphatase inhibitor cocktail), and lysates were subjected to immunoblotting. For all immunoblotting experiments, chemiluminescence was detected using a GE ImageQuant LAS 500 imager, and protein bands were quantified using Fiji software.

### In-gel digestion after pulldown

Coomassie-stained bands were cut out and washed with 200 µl of wash buffer (50 mM TEAB in 1:1 water:MeCN) until destained. In case of strongly colored bands, this process was repeated to fully remove Coomassie. The wash buffer was discarded; gel slices were equilibrated for 10 min at 30 °C in 50 mM TEAB; and the supernatant was discarded. Then, 200 µl of MeCN was added to shrink and dry gel pieces. This step was repeated once more.

IWS1 samples were then reduced and alkylated. NOLC1 and TCOF1, having no Cys residues, were not. Then, 100 µl of 5 mM DTT was added to each tube and incubated for 45 min at 56 °C, 300 r.p.m. on a shaker, and the supernatant was discarded. Next, 100 µl of 40 mM CAA in 50 mM TEAB was added and incubated for 30 min in the dark at room temperature, and the supernatant was discarded. Gel slices were incubated in 200 µl of wash buffer for 5 min at 30 °C, 300 r.p.m., and then in 200 µl of MeCN for 5 min. The MeCN step was repeated once more.

For digestion, 0.2 µg of trypsin in 30 µl of 50 mM TEAB was added to each gel slice. In some cases, more buffer was added until the gel piece was covered. Samples were incubated overnight at 37 °C and 300 r.p.m.

The next day, samples were briefly centrifuged, and digestion was stopped by adding 30 µl of 0.5% TFA in MeCN. The supernatant was transferred to an MS vial. Next, 20 µl of MeCN was added to the gel piece to shrink and dry it and then was added into the same vial. The combined solutions were dried in a vacuum centrifuge and stored at −20 °C until LC–MS analysis.

Samples were then redissolved in 10 µl of citrate injection buffer (50 mM sodium citrate, 3% MeCN) and sonicated for 5 min. Next, 2 µl was injected and analyzed in the same manner as the pyrophosphoproteomics samples described above.

### Protein pyrophosphorylation using [β^32^P]5PP-InsP_5_

[γ^32^P]ATP was procured from JONAKI/BRIT. Radiolabeled [β^32^P]5PP-InsP_5_ synthesis was conducted as described previously^68^, with a few modifications. In brief, for a 200-µl reaction, 200 µM InsP_6_ (SiChem) was incubated for 6 h at 37 °C with 3 mCi [γ^32^P]ATP and 50 µM unlabeled ATP in the presence of 80 ng µl^−1^ purified *Entamoeba histolytica* InsP_6_ kinase (IP6KA) in buffer containing 100 mM MES, pH 6.8, 30 mM MgSO_4_, 250 mM NaCl and 5 mM DTT. [β^32^P]5PP-InsP_5_ was purified by strong anion exchange HPLC (Waters) as described previously^68^.

Proteins were subjected to phosphorylation by CK2 and pyrophosphorylation by [β^32^P]5PP-InsP_5_ as described previously^16,20,31^. For in vitro pyrophosphorylation assays, immunoprecipitated proteins on beads were first pre-phosphorylated with CK2 (New England Biolabs) in protein kinase buffer (New England Biolabs) and 0.5 mM Mg^2+^-ATP for 30 min at 30 °C. Beads were washed in cold PBS and incubated in pyrophosphorylation buffer (25 mM HEPES, pH 7.4, 50 mM NaCl, 6 mM MgCl_2_, 1 mM DTT) containing 3–5-µCi [β^32^P]5PP-InsP_5_ at 37 °C for 15 min. Samples on beads were mixed with LDS sample buffer (Thermo Fisher Scientific), heated at 95 °C for 5 min, resolved on a 4–12% NuPAGE Bis-Tris gel (Thermo Fisher Scientific) and transferred to a PVDF membrane (GE Life Sciences). Pyrophosphorylation was detected using a phosphorimager (Typhoon FLA-9500), and proteins were detected by immunoblotting. To improve visualization, the phosphorimager scan and immunoblots were subjected to uniform ‘Levels’ adjustment in Adobe Photoshop.

Back-pyrophosphorylation was conducted as described previously^19–21^, and the method is explained schematically in Fig. 5b. Overexpressed or endogenous UBF1 was immunoprecipitated from *IP6K1*^−/−^ HEK293T cells expressing either active or kinase-dead IP6K1. The immunoprecipitated proteins were subjected to pyrophosphorylation as described above. Radiolabeled protein as a fraction of total immunoprecipitated protein was quantified using Fiji software^69^.

### RT–qPCR

Cells were lysed using TRIzol reagent (Thermo Fisher Scientific), and total RNA was extracted using a kit (HiMedia). Where indicated, cells were treated with 10 µM TNP^51^ (Merck Millipore), 1 µM SC-919 (synthesized as described by Kröber et al.^70^) or DMSO for 5 h before RNA isolation. cDNA was prepared by reverse transcription with SuperScript Reverse Transcriptase III (Thermo Fisher Scientific) using random hexamers. Two sets of 45S pre-rRNA specific primers were designed to amplify the region between sites 01 (also called A′) and A0 on the human 47S pre-rRNA transcript^71^. GAPDH transcript was used as an internal control. The sequences of the primers are provided in Supplementary Table 7. qPCR was conducted with SYBR Green PCR Master Mix on a CFX96 Touch Real-Time PCR Detection System (Bio-Rad). All samples were run in technical duplicates. The fold change (ΔΔC_T_) method^72^ was used to calculate the difference in transcript levels. ΔC_T_ refers to the C_T_ value for the target pre-rRNA normalized to the C_T_ value for GAPDH in the same sample. ΔΔC_T_ values were determined as a relative change in ΔC_T_ in HCT116 DKO compared to WT cells or drug-treated compared to control cells. 2^−ΔΔC^_T_ was used to represent fold changes.

### Statistical analysis

Statistical analyses and graph preparation were done using GraphPad Prism 8 software. The number of independent experimental replicates for each analysis or image is provided in the respective figure legend. Quantified data are presented as mean ± s.e.m. for the indicated number of biological replicates (*n*). *P* values were calculated using a one-sample *t*-test. *P* ≤ 0.05 was considered statistically significant.

### Reporting summary

Further information on research design is available in the Nature Portfolio Reporting Summary linked to this article.

## Online content

Any methods, additional references, Nature Portfolio reporting summaries, source data, extended data, supplementary information, acknowledgements, peer review information; details of author contributions and competing interests; and statements of data and code availability are available at 10.1038/s41589-024-01613-5.

## Supplementary information

Supplementary Information Supplementary Figs. 1–5.

Reporting Summary

Supplementary Table 1 MS results; pyrophosphoproteomics on a biological triplicate of HEK293T cells.

Supplementary Table 2 MS results of HEK293T replicate 1 using a decoy search algorithm instead of the standard workflow.

Supplementary Table 3 List of all discovered pyrophosphorylation sites.

Supplementary Table 4 Raw results of the Gene Ontology search from Fig. 3.

Supplementary Table 5 Raw MS results for the MS experiment with purified protein samples shown in Fig. 5.

Supplementary Table 6 MS results; pyrophosphoproteomics on a single biological replicate of *PPIP5K*^−/−^ HEK293T cells.

Supplementary Table 7 List of oligonucleotides (Table 7) and antibodies (Table 8) used in this work.

## Source data

Source Data Fig. 4 Unprocessed blots and gels.

Source Data Fig. 4 Numerical source data for the graph in Fig. 4d.

Source Data Fig. 5 Unprocessed blots and gels.

Source Data Fig. 5 Numerical source data for blot intensity analysis in Fig. 5c,d and qRT–PCR experiments in Fig. 5e–g.

Source Data Extended Data Fig. 5 Unprocessed blots and gels.

Source Data Extended Data Fig. 5 Numerical source data for Extended Data Fig. 5b,c.

## Data Availability

The reported proteomics data are publicly available on the jPOST repository^73^ under accession numbers JPST001935 / PXD038962, JPST001934 / PXD038963 and JPST002429 / PXD048031. Source data are provided with this paper.
