# Supplementary material for: Extensive protein pyrophosphorylation revealed in human cell lines
Source: Nat Chem Biol. 2024 Apr 25;20(10):1305–16. doi: 10.1038/s41589-024-01613-5 (PMC11427299; doi:10.1038/s41589-024-01613-5)
Supplement: Supplementary file 1 — Supplementary Figs. 1–5. [file 41589_2024_1613_MOESM1_ESM.pdf]

nature chemical biology

Article https://doi.org/10.1038/s41589-024-01613-5

Extensive protein pyrophosphorylation revealed in human cell lines

In the format provided by the authors and unedited

Contents:

Supplementary Figure 1: Distribution of PSMs over fractions 1-12 after hSAX fractionation

Supplementary Figure 2: Screenshots illustrating the process of manual pyrophosphosite validation.

Supplementary Figure 3: Detailed annotation of spectra from Fig. 2

Supplementary Figure 4: UBF1 sequence composition in comparison with TCOF1, NOLC1 and IWS1.

Supplementary Figure 5: Extended analysis of Figure 5a

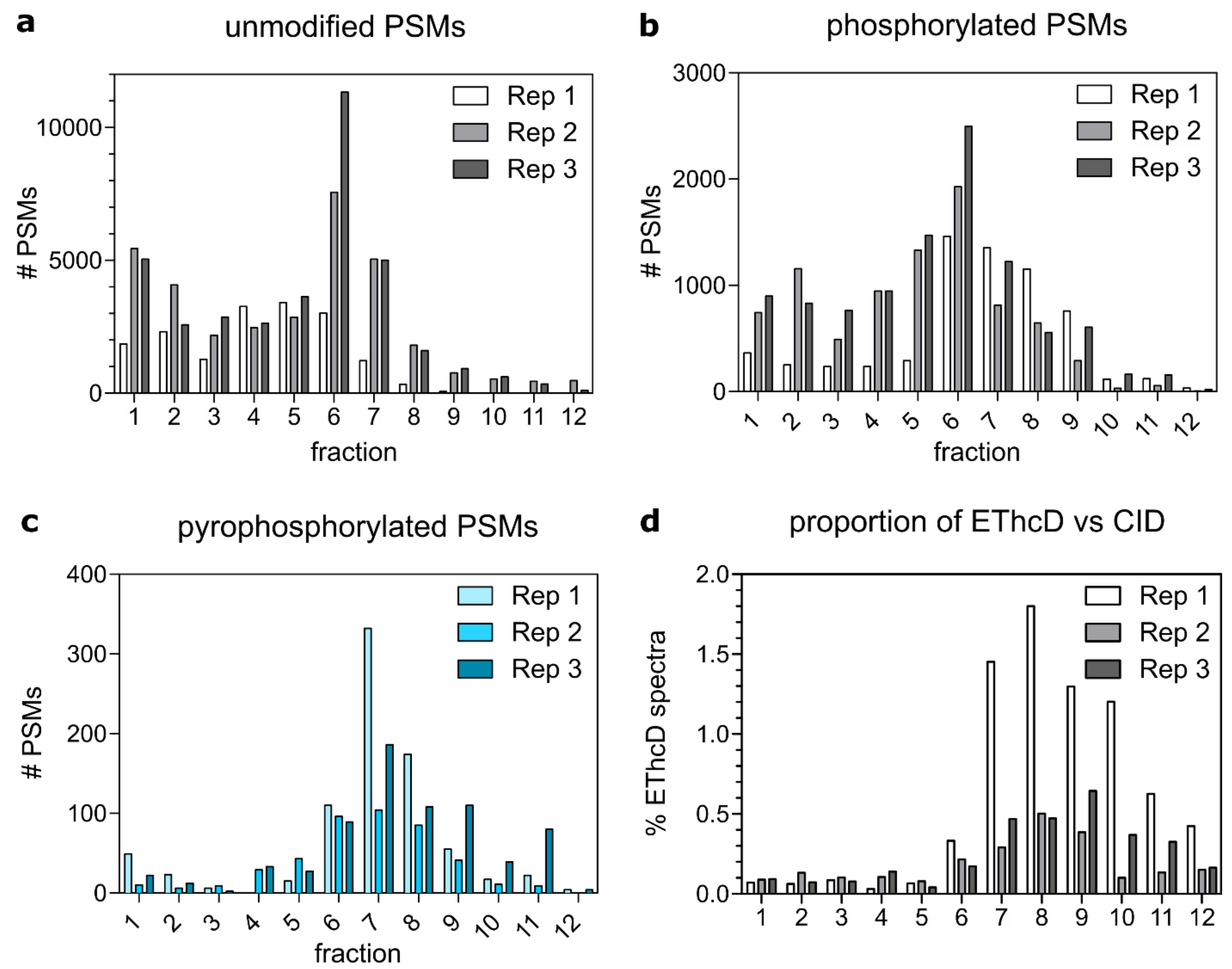

Supplementary Figure 1: Distribution over fractions 1-12 after hSAX fractionation of PSMs annotated as a) pyrophosphorylated / b) phosphorylated / c) neither by the SequestHT search engine. d) Proportion of EThcD spectra vs. CID spectra. Three biological replicates from HEK292T were analyzed.

a

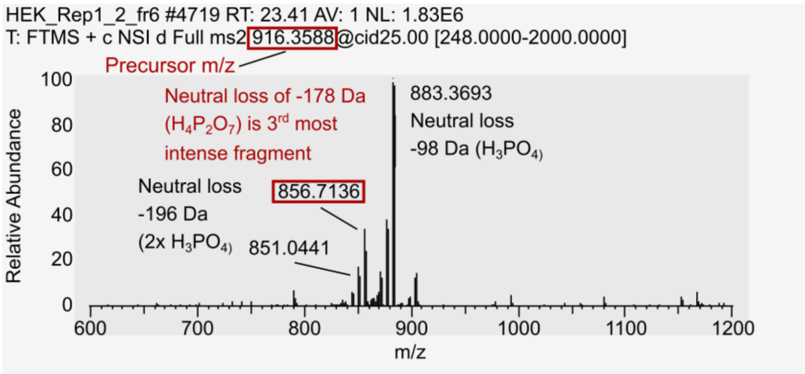

b

#	Immon.	b	c	Seq.
1	44.050			A*
2	44.050	223.048	240.075	A
3	74.061	324.096	341.122	T
4	74.061	425.143	442.170	T
5	70.066	522.196	539.223	P
6	74.061	623.244	640.270	T
7	129.114	779.345	796.372	R
8	101.108	907.440	924.466	K
9	60.045	994.472	1011.498	S
10	60.045	1081.504	1098.531	S
11	60.045	1168.536	1185.563	S
12	60.045	1255.568	1272.595	S
13	102.056	1384.611	1401.637	E
14	88.040	1499.638	1516.664	D
15	60.045	1586.670	1603.696	S
16	60.045	1673.702	1690.728	S
17	60.045	1760.734	1777.760	S
18	88.040	1875.761	1892.787	D
19	102.056	2004.803	2021.830	E
20	102.056	2133.846	2150.872	E
21	102.056	2262.888	2279.915	E
22	102.056	2391.931	2408.958	E
23	101.071	2519.990	2537.016	Q
24	101.108			K

#	Immon.	b	c	Seq.	y	z	#
1	44.050			A	2746.061	2730.042	24
2	44.050	143.082	160.109	A	2675.023	2659.004	23
3	74.061	244.130	261.156	T	2603.986	2587.967	22
4	74.061	345.177	362.204	T	2502.938	2486.919	21
5	70.066	442.230	459.257	P	2401.890	2385.872	20
6	74.061	543.278	560.304	T	2304.838	2288.819	19
7	129.114	699.379	716.406	R	2203.790	2187.771	18
8	101.108	827.474	844.500	K	2047.689	2031.670	17
9	60.045	1074.438	1091.464	S**	1919.594	1903.575	16
10	60.045	1161.470	1178.496	S	1672.630	1656.611	15
11	60.045	1248.502	1265.529	S	1585.598	1569.579	14
12	60.045	1335.534	1352.561	S	1498.566	1482.547	13
13	102.056	1464.577	1481.603	E	1411.534	1395.515	12
14	88.040	1579.604	1596.630	D	1282.491	1266.472	11
15	60.045	1666.636	1683.662	S	1167.464	1151.445	10
16	60.045	1753.668	1770.694	S	1080.432	1064.413	9
17	60.045	1840.700	1857.726	S	993.400	977.381	8
18	88.040	1955.727	1972.753	D	906.368	890.349	7
19	102.056	2084.769	2101.796	E	791.341	775.322	6
20	102.056	2213.812	2230.838	E	662.299	646.280	5
21	102.056	2342.854	2359.881	E	533.256	517.237	4
22	102.056	2471.897	2488.924	E	404.213	388.195	3
23	101.071	2599.956	2616.982	Q	275.171	259.152	2
24	101.108			K	147.112	131.094	1

Seq.	y	z	#
A	2666.094	2650.076	24
A	2595.057	2579.038	23
T	2524.020	2508.001	22
T	2422.972	2406.953	21
P	2321.924	2305.906	20
T	2224.872	2208.853	19
R	2123.824	2107.805	18
K	1967.723	1951.704	17
S	1839.628	1823.609	16
S	1752.596	1736.577	15
S	1665.564	1649.545	14
S	1578.532	1562.513	13
E	1491.500	1475.481	12
D	1362.457	1346.438	11
S	1247.430	1231.411	10
S	1160.398	1144.380	9
S	1073.366	1057.347	8
D	986.334	970.315	7
E	871.307	855.288	6
E	742.265	726.246	5
E	613.222	597.203	4
E	484.179	468.161	3
Q	355.137	339.118	2
K*	227.078	211.060	1

c

#1	b+	c+	Seq.	y+	z+	#2
1	72.04439	89.07094	A			24
2	143.08150	160.10805	A	2675.02408	2659.00536	23
3	244.12918	261.15573	T	2603.98697	2587.96824	22
4	345.17686	362.20341	T	2502.93929	2486.92056	21
5	442.22962	459.25617	P	2401.89161	2385.87289	20
6	543.27730	560.30385	T	2304.83885	2288.82012	19
7	699.37841	716.40496	R	2203.79117	2187.77244	18
8	827.47338	844.49993	K	2047.69006	2031.67133	17
9	1074.43807	1091.46462	S-pyropho...	1919.59509	1903.57637	16
10	1161.47010	1178.49664	S	1672.63040	1656.61168	15
11	1248.50212	1265.52867	S	1585.59837	1569.57965	14
12	1335.53415	1352.56070	S	1498.56635	1482.54762	13
13	1464.57675	1481.60329	E	1411.53432	1395.51559	12
14	1579.60369	1596.63024	D	1282.49172	1266.47300	11
15	1666.63572	1683.66227	S	1167.46478	1151.44606	10
16	1753.66775	1770.69429	S	1080.43275	1064.41403	9
17	1840.69977	1857.72632	S	993.40073	977.38200	8
18	1955.72672	1972.75327	D	906.36870	890.34997	7
19	2084.76931	2101.79586	E	791.34175	775.32303	6
20	2213.81190	2230.83845	E	662.29916	646.28044	5
21	2342.85450	2359.88104	E	533.25657	517.23784	4
22	2471.89709	2488.92364	E	404.21397	388.19525	3
23	2599.95567	2616.98222	Q	275.17138	259.15266	2
24			K	147.11280	131.09408	1

#1	b+	c+	Seq.	y+	z+	#2
1	72.04439	89.07094	A			24
2	143.08150	160.10805	A	2675.02408	2659.00536	23
3	244.12918	261.15573	T	2603.98697	2587.96824	22
4	345.17686	362.20341	T	2502.93929	2486.92056	21
5	442.22962	459.25617	P	2401.89161	2385.87289	20
6	543.27730	560.30385	T	2304.83885	2288.82012	19
7	699.37841	716.40496	R	2203.79117	2187.77244	18
8	827.47338	844.49993	K	2047.69006	2031.67133	17
9	994.47174	1011.49829	S-Phospho	1919.59509	1903.57637	16
10	1161.47010	1178.49664	S-Phospho	1752.59673	1736.57801	15
11	1248.50212	1265.52867	S	1585.59837	1569.57965	14
12	1335.53415	1352.56070	S	1498.56635	1482.54762	13
13	1464.57675	1481.60329	E	1411.53432	1395.51559	12
14	1579.60369	1596.63024	D	1282.49172	1266.47300	11
15	1666.63572	1683.66227	S	1167.46478	1151.44606	10
16	1753.66775	1770.69429	S	1080.43275	1064.41403	9
17	1840.69977	1857.72632	S	993.40073	977.38200	8
18	1955.72672	1972.75327	D	906.36870	890.34997	7
19	2084.76931	2101.79586	E	791.34175	775.32303	6
20	2213.81190	2230.83845	E	662.29916	646.28044	5
21	2342.85450	2359.88104	E	533.25657	517.23784	4
22	2471.89709	2488.92364	E	404.21397	388.19525	3
23	2599.95567	2616.98222	Q	275.17138	259.15266	2
24			K	147.11280	131.09408	1

Supplementary Figure 2: Screenshots illustrating the process of manual pyrophosphosite validation. a) Screenshot from “Freestyle” showing the CID spectrum of a putative pyrophosphopeptide (NOLC1 556-279). The fragment peak corresponding to the neutral loss of pyrophosphoric acid is the third most intense peak, meeting the requirements. b) Screenshot from the “Peptide Sequence Fragmentation Modeling” window of “Molecular Weight Calculator”. The panel in the middle shows the overlap (turquoise fields) between theoretical and experimentally observed EThcD fragments of the peptide from a), which is pyrophosphorylated in position 9 (Ser264). In the panels to the left and right, a hypothetical phosphoryl group has been added to the C- or N-terminus of the sequence (red boxes), to calculate the masses of all possible monophosphorylated fragments. None of these masses have been found in the EThcD spectrum, so that all fields remain grey. c) Screenshots from “Proteome Discoverer” showing overlap between the fragment ions in an EThcD spectrum of the precursor from a) and theoretical fragments of two different PSMs. The red and blue fragment masses have been found in the spectrum. Left panel: Pyrophosphopeptide from b). The fragmentation covers the putative pyrophosphoserine and the adjacent phosphorylatable residues. Right panel: Bisphosphopeptide phosphorylated in position 9 and 10. Both PSMs were assigned a localization probability of 1.0 by ptmRS, but manual inspection reveals that key fragments for the bisphospho-PSM are missing.

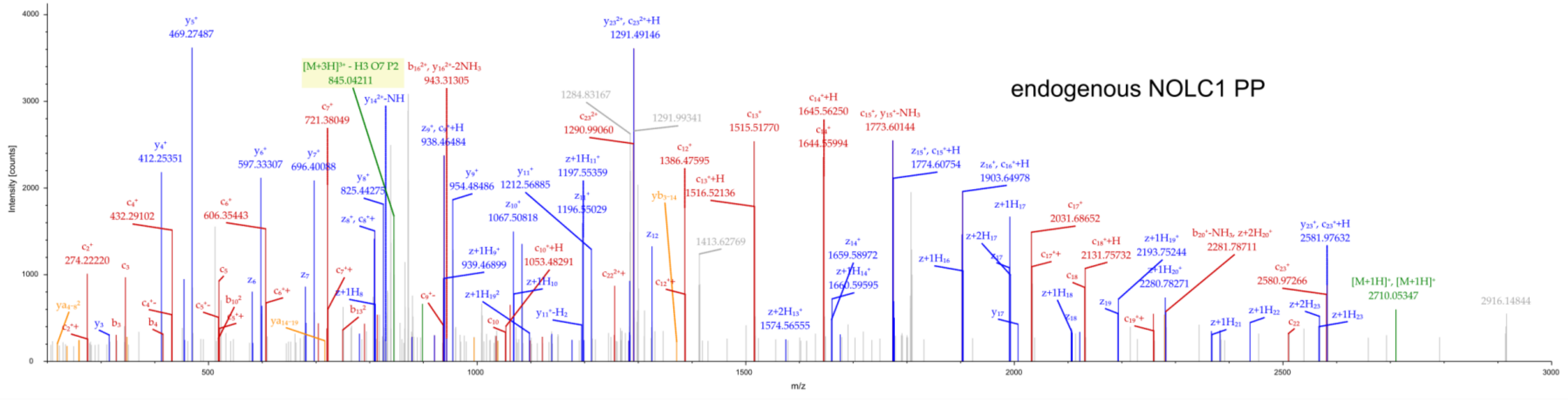
endogenous NOLC1 PP
Intensity [counts]
m/z

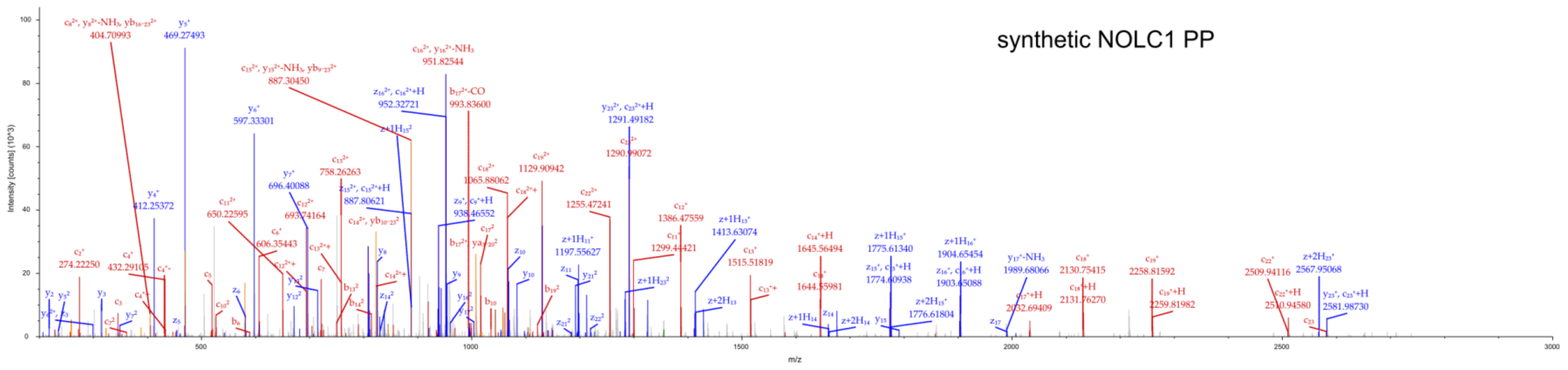
synthetic NOLC1 PP
Intensity [counts] (10^3)
m/z

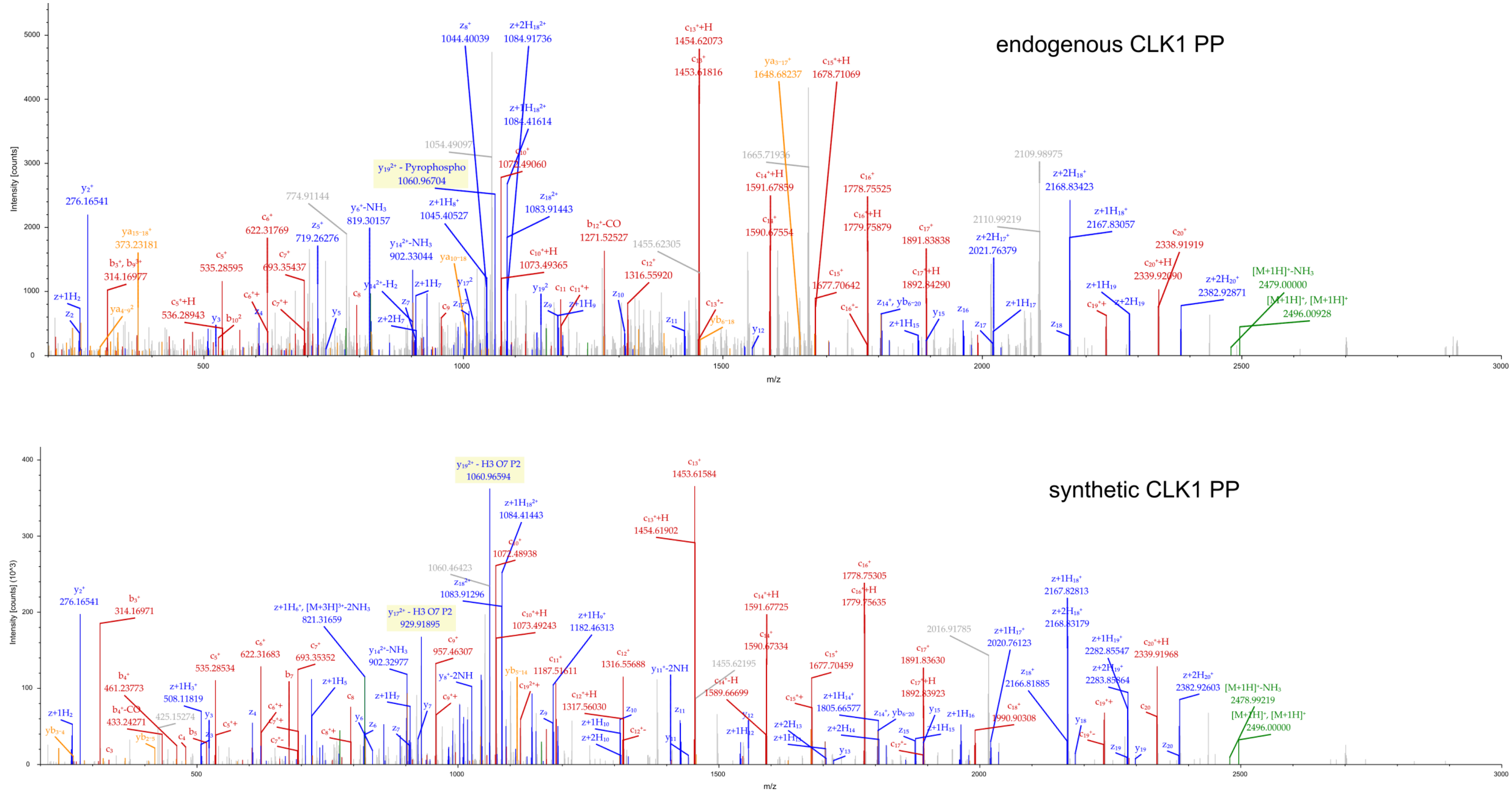

Supplementary Figure 3: Detailed annotation of spectra from Fig. 2

.

a

Treacher Collins syndrome protein 1 (TCOF1)

```
        10         20         30         40         50
MAEARKRREL LPLIYHHLLR AGYVRAAREV KEQSGQKCFL AQPVTLLDIY
        60         70         80         90        100
THWQQTSELG RKRKAEEDAA LQAKKTRVSD PISTSESSEE EEEAEAETAK
       110        120        130        140        150
ATPRLASTNS SVLGADLPSS MKEKAKAETE KAGKTGNSMP HPATGKTVAN
       160        170        180        190        200
LLSGKSPRKS AEPSANTTLV SETEEEGSVP AFGAAAKPGM VSAGQADSSS
       210        220        230        240        250
EDTSSSSDET DVEGKPSVKP AQVKASSVST KESPARKAAP APGKVGDVTP
       260        270        280        290        300
QVKGGALPPA KRAKKPEEES ESSEEGSESE EEAPAGTRSQ VKASEKILQV
       310        320        330        340        350
RAASAPAKGT PGKGATPAPP GKAGAVASQT KAGKPEEDSE SSSEESSDSE
       360        370        380        390        400
EETPAAKALL QAKASGKTSQ VGAASAPAKE SPRKGAAPAP PGKTGPAVAK
       410        420        430        440        450
AQAGKREEDS QSSSEESDSE EEAPAQAKPS GKAPQVRAAS APAKESPRKG
       460        470        480        490        500
AAPAPPRKTG PAAAQVQVGK QEEDSRSSSE ESDSDREALA AMNAAQVKPL
       510        520        530        540        550
GKSPQVKPAS TMGMGPLGKG AGPVPPGKVG PATPSAQVGK WEEDSESSSE
       560        570        580        590        600
ESSDSSDGEV PTAVAPAQEK SLGNILQAKP TSSPAKGPPQ KAGPVAVQVK
       610        620        630        640        650
AEKPMDNSES SEESSDSADS EEAPAAMTAA QAKPALKIPQ TKACPKKTNT
       660        670        680        690        700
TASAKVAPVR VGTQAPRKAG TATSPAGSSP AVAGGTQRPA EDSSSSEESD
       710        720        730        740        750
SEEEKTGLAV TVGQAKSVGK GLQVKAASVP VKGSLGQGTA PVLPGKTGPT
       760        770        780        790        800
VTQVKAEKQE DSESSEEESD SEEAAASPAQ VKTSVKKTQA KANPAAARAP
       810        820        830        840        850
SAKGTISAPG KVVTAAAQAK QRSPSKVKPP VRNPQNSTVL ARGPASVPSV
       860        870        880        890        900
GKAVATAAQA QTGPEEDSGS SEEESDSEEE AETLAQVKPS GKTHQIRAAL
       910        920        930        940        950
APAKESPRKG AAPTPPGKTG PSAAQAGKQD DSGSSSEESD SDGEAPAAVT
       960        970        980        990       1000
SAQVIKPPLI FVDPNRSPAG PAATPAQAQA ASTPRKARAS ESTARSSSSE
      1010       1020       1030       1040       1050
SEDEDVIPAT QCLTPGIRTN VVTMPTAHPR IAPKASMAGA SSSKESSRIS
      1060       1070       1080       1090       1100
DGKKQEGPAT QVSKKNPASL PLTQAALKVL AQKASEAQPP VARTQPSSGV
      1110       1120       1130       1140       1150
DSAVGTLPAT SPQSTSVQAK GTNKLRKPKL PEVQQATKAP ESSDDSEDSS
      1160       1170       1180       1190       1200
DSSSGSEEDG EGPQGAKSAH TLGPTPSRTE TLVEETAAES SEDDVVAPSQ
      1210       1220       1230       1240       1250
SLLSGYMTPG LTPANSQASK ATPKLDSSPS VSSTLAAKDD PDGKQEAKPQ
      1260       1270       1280       1290       1300
QAAGMLSPKT GGKEAASGTT PQKSRKPKKG AGNPQASTLA LQSNITQCLL
      1310       1320       1330       1340       1350
GQPWPLNEAQ VQASVVKVLT ELLEQERKKV VDTTKESSRK GWESRKRKLS
      1360       1370       1380       1390       1400
GDQPAARTPR SKKKKKLGAG EGGEASVSPE KTSTTSKGKA KRDKASGDVK
      1410       1420       1430       1440       1450
EKKGKGSLGS QGAKDEPEEE LQKGMGTVEG GDQSNPKSKK EKKKSDKRKK
      1460       1470       1480
DKEKKEKKKK AKKASTKDSE SPSQKKKKKK KKTAEQTV
```

Nucleolar and coiled-body phosphoprotein 1 (NOLC1)

```
        10         20         30         40         50
MADAGIRRVV PSDLYPLVLG FLRDNQLSEV ANKFAKATGA TQQDANASSL
        60         70         80         90        100
LDIYSFWLKS AKVPERKLQA NGPVAKKAKK KASSSDSEDS SEEEEEVQGP
       110        120        130        140        150
PAKKAAVPAK RVGLPPGKAA AKASESSSSE ESSDDDDEED QKKQPVQKGV
       160        170        180        190        200
KPQAKAAKAP PKKAKSSDSD SDSSSEDEPP KNQKPKITPV TVKAQTKAPP
       210        220        230        240        250
KPARAAPKIA NGKAASSSSS SSSSSSDDS EEEKAAATPK KTVPKKQVVA
       260        270        280        290        300
KAPVKAATTP TRKSSSSEDS SSDEEEEQKK PMKNKPGPYS SVPPPSAPPP
       310        320        330        340        350
KKSLGTQPPK KAVEKQQPVE SSEDSSDESD SSSEEEKKPP TKAVVSKATT
       360        370        380        390        400
KPPPAKKAAE SSSDSSDSDS SEDDEAPSKP AGTTKNSSNK PAVTTKSPAV
       410        420        430        440        450
KPAAAPKQPV GGGQKLLTRK ADSSSSEEES SSSEEEKTKK MVATTKPKAT
       460        470        480        490        500
AKAALSLPAK QAPQGSRDSS SDSDSSSSEE EEEKTSKSAV KKKPQKVAGG
       510        520        530        540        550
AAPSKPASAK KGKAESSNSS SSDDSSEEEE EKLKGKGSPR PQAPKANGTS
       560        570        580        590        600
ALTAQNGKAA KNSEEEEEEK KKAAVVVSKS GSLKKRKQNE AAKEAETPQA
       610        620        630        640        650
KKIKLQTPNT FPKRKKGEKR ASSPFRRVRE EEIEVDSRVA DNSFDAKRGA
       660        670        680        690
AGDWGERANQ VLKFTKGKSF RHEKTKKKRG SYRGGSISVQ VNSIKFDSE
```

IWS1

```
        10         20         30         40         50
MDSEYYSGDQ SDDGGATPVQ DERDSGSDGE DDVNEQHSGS DTGSVERHSE
        60         70         80         90        100
NETSDREDGL PKGHHVTDSE NDEPLNLNAS DSESEELHRQ KDSDSESEER
       110        120        130        140        150
AEPPASDSEN EDVNQHGSDS ESEETRKLPG SDSENEELLN GHASDSENED
       160        170        180        190        200
VGKHPASDSE IEELQKSPAS DSETEDALKP QISDSESEEP PRHQASDSEN
       210        220        230        240        250
EEPPKPRMSD SESEELPKPQ VSDSESEEPP RHQASDSENE ELPKPRISDS
       260        270        280        290        300
ESEDPPRHQA SDSENEELPK PRISDSESED PPRNQASDSE NEELPKPRVS
       310        320        330        340        350
DSESEGPQKG PASDSETEDA SRHKQKPESD DDSDRENKGE DTEMQNDSFH
       360        370        380        390        400
SDSHMDRKKF HSSDSEEEEH KKQKMDSDED EKEGEEEKVA KRKAAVLSDS
       410        420        430        440        450
EDEEKASAKK SRVVSDADDS DSDAVSDKSG KREKTIASDS EEEAGKELSD
       460        470        480        490        500
KKNEEKDLFG SDSESGNEEE NLIADIFGES GDEEEEEFTG FNQEDLEEEK
       510        520        530        540        550
GETQVKEAED SDSDDNIKRG KHMDFLSDFE MMLQRKKSMS GKRRRNRDGG
       560        570        580        590        600
TFISDADDVV SAMIVKMNEA AEEDRQLNNQ KKPALKKLTL LPAVVMHLKK
       610        620        630        640        650
QDLKETFIDS GVMSAIKEWL SPLPDRSLPA LKIREELLKI LQELPSVSQE
       660        670        680        690        700
TLKHSGIGRA VMYLYKHPKE SRSNKDMAGK LINEWSRPIF GLTSNYKGMT
       710        720        730        740        750
REEREQRDLE QMPQRRRMNS TGGQTPRRDL EKVLTGEEKA LRPGDPGFCA
       760        770        780        790        800
RARVPMPSNK DYVVRPKWNV EMESSRFQAT SKKGISRLDK QMRKFTDIRK
       816
KSRSAHAVKI SIEGNKMPL
```

b

Upstream binding factor 1 (UBF1)

```
        10         20         30         40         50
MNGEADCPTD LEMAAPKGQD RWSQEDMLTL LECMKNNLPS NDSSKFKTTE
        60         70         80         90        100
SHMDWEKVAF KDFSGDMCKL KWVEISNEVR KFRTLTELIL DAQEHVKNPY
       110        120        130        140        150
KGKKLKKHPD FPKKPLTPYF RFFMEKRAKY AKLHPEMSNL DLTKILSKKY
       160        170        180        190        200
KELPEKKKMK YIQDFQREKQ EFERNLARFR EDHPDLIQNA KKSDIPEKPK
       210        220        230        240        250
TPQQLWYTHE KKVYLKVRPD ATTKEVKDSL GKQWSQLSDK KRLKWIHKAL
       260        270        280        290        300
EQRKEYEEIM RDYIQKHPEL NISEEGITKS TLTKAERQLK DKFDGRPTKP
       310        320        330        340        350
PPNSYSLYCA ELMANMKDVP STERMVLCSQ QWKLLSQKEK DAYHKKCDQK
       360        370        380        390        400
KKDYEVELLR FLESLPEEEQ QRVLGEEKML NINKKQATSP ASKKPAQEGG
       410        420        430        440        450
KGGSEKPKRP VSAMFIFSEE KRRQLQEERP ELSESELTRL LARMWNDLSE
       460        470        480        490        500
KKKAKYKARE AALKAQSERK PGGEREERGK LPESPKRAEE IWQQSVIGDY
       510        520        530        540        550
LARFKNDRVK ALKAMEMTWN NMEKKEKLMW IKKAAEDQKR YERELSEMRA
       560        570        580        590        600
PPAATNSSKK MKFQGEPKKP PMNGYQKFSQ ELLSNGELNH LPLKERMVEI
       610        620        630        640        650
GSRWQRISQS QKEHYKKLAE EQQKQYKVHL DLWVKSLSPQ DRAAYKEYIS
       660        670        680        690        700
NKRKSMTKLR GPNPKSSRTT LQSKSESEED DEEDEDDEDE DEEEEDDENG
       710        720        730        740        750
DSSEDGGDSS ESSSEDESED GDENEEDDED EDDDEDDDED EDNESEGSSS
       760
SSSSSGDSSD SDSN
```

Supplementary Figure 4: UBF1 sequence composition in comparison with TCOF1, NOLC1 and IWS1. The predicted IDRs on TCOF1, NOLC1, IWS1 and UBF1 are indicated with a light blue highlight. The ppSer residues identified in TCOF1, NOLC1, and IWS1 are in turquoise, and the predicted ppSer residues in UBF1 are in blue. Lys or Arg residues marking the trypsin cleavage sites of the pyrophosphopeptides identified in TCOF1, NOLC1, and IWS1 are in red, and the trypsin cleavage sites neighboring the predicted pyrophosphosites in UBF1 are in magenta. The identified tryptic pyrophosphopeptides in TCOF1, NOLC1, and IWS1 are underlined, and the predicted pyrophosphopeptides in UBF1 are indicated with a dotted line. IuPred2A was used to predict regions of disorder in all four proteins, and Scansite 4.0 was used to predict pre-phosphorylation sites on UBF1.

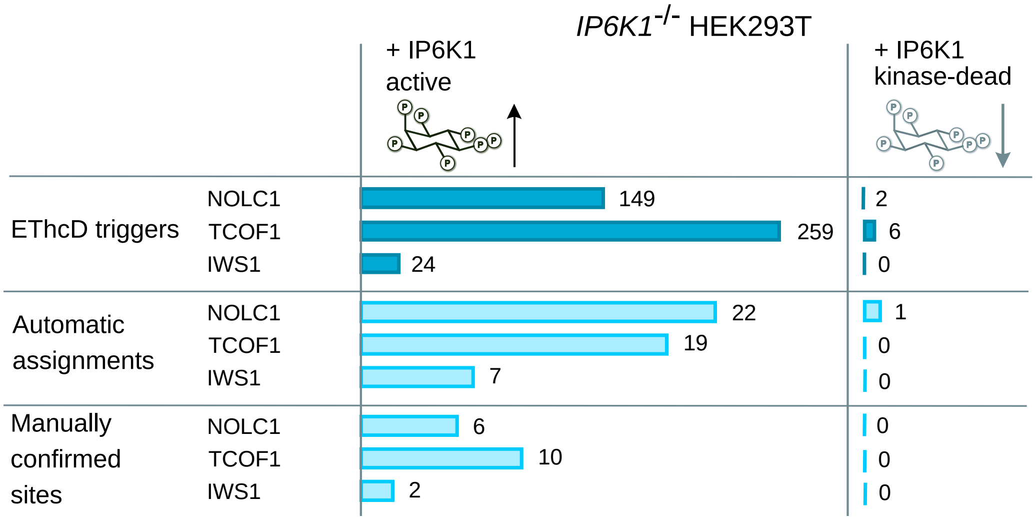

Supplementary Figure 5: Extended analysis of Figure 5a. Comparative MS analysis of pyrophosphosites detected in SFB-tagged NOLC1, TCOF1 or IWS1 co-expressed with V5-tagged active or kinase-dead IP6K1 (left). Number of neutral-loss triggered EThcD spectra vs pyrophosphopeptides assigned by automatic or manual analysis. EThcD triggers were monitored as an estimate of pyrophosphorylation abundance. The reduction in EThcD triggers correlated well with the reduction in automatic and manually assigned sites, indicating both the number of assigned sites and overall abundance of the modification appear to be PP-InsP-dependent.
