# Supplementary material for: Extensive protein pyrophosphorylation revealed in human cell lines
Source: Nat Chem Biol. 2024 Apr 25;20(10):1305–16. doi: 10.1038/s41589-024-01613-5 (PMC11427299; doi:10.1038/s41589-024-01613-5)
Supplement: Supplementary file 2 — Reporting Summary [file 41589_2024_1613_MOESM2_ESM.pdf]

nature portfolio

Corresponding author(s): Rashna Bhandari and Dorothea Fiedler

Last updated by author(s): Mar 12, 2024

Reporting Summary

Nature Portfolio wishes to improve the reproducibility of the work that we publish. This form provides structure for consistency and transparency in reporting. For further information on Nature Portfolio policies, see our Editorial Policies and the Editorial Policy Checklist.

Statistics

For all statistical analyses, confirm that the following items are present in the figure legend, table legend, main text, or Methods section.

n/a	Confirmed	
☐	☒	The exact sample size (*n*) for each experimental group/condition, given as a discrete number and unit of measurement
☐	☒	A statement on whether measurements were taken from distinct samples or whether the same sample was measured repeatedly
☐	☒	The statistical test(s) used AND whether they are one- or two-sided<br>*Only common tests should be described solely by name; describe more complex techniques in the Methods section.*
☒	☐	A description of all covariates tested
☒	☐	A description of any assumptions or corrections, such as tests of normality and adjustment for multiple comparisons
☐	☒	A full description of the statistical parameters including central tendency (e.g. means) or other basic estimates (e.g. regression coefficient) AND variation (e.g. standard deviation) or associated estimates of uncertainty (e.g. confidence intervals)
☐	☒	For null hypothesis testing, the test statistic (e.g. F, t, r) with confidence intervals, effect sizes, degrees of freedom and P value noted<br>*Give P values as exact values whenever suitable.*
☒	☐	For Bayesian analysis, information on the choice of priors and Markov chain Monte Carlo settings
☒	☐	For hierarchical and complex designs, identification of the appropriate level for tests and full reporting of outcomes
☒	☐	Estimates of effect sizes (e.g. Cohen's d, Pearson's r), indicating how they were calculated

Our web collection on statistics for biologists contains articles on many of the points above.

Software and code

Policy information about availability of computer code

Data collection	The Orbitrap Fusion mass spectrometer was operated using the Xcalibur software package (Thermofisher Scientific) version 4.4.16.14. 3500xL Genetic Analyzer (Applied Biosystems) was used for the genotyping of IP6K1-/- HEK293T cell line. For immunofluorescence, images were taken on Leica TCS SP8 confocal microscope using LAS X software version 3.7.4.23463 or Elyra 7 structured illumination microscopy (SIM) module of the Zeiss LSM 980 confocal microscope using ZEN software version 3.2. RT-qPCR data were collected on a CFX96 Touch Real-Time PCR Detection System (Biorad) using CFX Maestro software version 1.1. Autoradiographs were detected using a phosphorimager Typhoon FLA-9500 (GE Healthcare). Chemiluminescence was detected using the GE ImageQuant LAS 500 imager (GE Healthcare).
Data analysis	MS raw data was analyzed using Proteome Discoverer (Thermofisher Scientific) version 2.4.0.305, FreeStyle (Thermofisher Scientific) version 1.7.73.12, Molecular Weight Calculator (Matthew Monroe https://alchemistmatt.com/resume/mwtoverview.html) version 6.50 and MaxQuant version 2.0.3.0. IuPred2A was used for disorder prediction. Scansite 4.0 was used to predict motifs that are likely to undergo phosphorylation by specific protein kinases. Gene ontology term analysis was done using Enrichr. Radiolabeled protein as a fraction of total immunoprecipitated protein was quantified using Fiji software version 1.53t. To improve visualization, the phosphorimager scan and immunoblots were subjected to uniform "Levels" adjustment in Adobe Photoshop version 10.0. Statistical analyses and graph preparation were done using GraphPad Prism 8.

For manuscripts utilizing custom algorithms or software that are central to the research but not yet described in published literature, software must be made available to editors and reviewers. We strongly encourage code deposition in a community repository (e.g. GitHub). See the Nature Portfolio guidelines for submitting code & software for further information.

Data

Policy information about availability of data

All manuscripts must include a data availability statement. This statement should provide the following information, where applicable:

- Accession codes, unique identifiers, or web links for publicly available datasets
- A description of any restrictions on data availability
- For clinical datasets or third party data, please ensure that the statement adheres to our policy

The reported proteomics data is publicly available on the jPOST repository under the accession numbers: JPST001935 / PXD038962, JPST001934 / PXD038963 and JPST002429 / PXD048031. Source Data are provided with this paper.

Human research participants

Policy information about studies involving human research participants and Sex and Gender in Research.

Reporting on sex and gender	not applicable
Population characteristics	not applicable
Recruitment	not applicable
Ethics oversight	not applicable

Note that full information on the approval of the study protocol must also be provided in the manuscript.

Field-specific reporting

Please select the one below that is the best fit for your research. If you are not sure, read the appropriate sections before making your selection.

☒ Life sciences ☐ Behavioural & social sciences ☐ Ecological, evolutionary & environmental sciences

For a reference copy of the document with all sections, see nature.com/documents/nr-reporting-summary-flat.pdf

Life sciences study design

All studies must disclose on these points even when the disclosure is negative.

Sample size	Sample sizes were chosen based on our prior extensive experience on the assays performed in this study (Thota et al., Biochemical J 2015; Chanduri et al., Biochemical Journal 2016; Lolla et al., Biochemical Journal 2021; Jadav et al., Journal of Biological Chemistry, 2013; Jadav et al., Cellular Signalling, 2016; Shah and Bhandari, Journal of Cell Science, 2021)
Data exclusions	No data was excluded from the analysis.
Replication	As per standard practices in Cell biology and Biochemistry, experiments were conducted in independent biological replicates. The number of replicates in each dataset is indicated in the respective figure Legend. All attempts at replication were successful.
Randomization	Cell culture plates were randomly assigned for treatment with vehicle control or IP6K inhibitor. In other cell-based experiments including immunofluorescence, western blotting, protein pyrophosphorylation, and mass spectrometry, randomization is not possible or applicable.
Blinding	Western blot analysis was not blinded as the gel loading order needs to be defined. Immunofluorescence analysis did not need blinding as separate groups were not compared. For MS experiments, blinding is not applicable.

Reporting for specific materials, systems and methods

We require information from authors about some types of materials, experimental systems and methods used in many studies. Here, indicate whether each material, system or method listed is relevant to your study. If you are not sure if a list item applies to your research, read the appropriate section before selecting a response.

Materials & experimental systems

n/a	Involved in the study	
☐	☒	Antibodies
☐	☒	Eukaryotic cell lines
☒	☐	Palaeontology and archaeology
☒	☐	Animals and other organisms
☒	☐	Clinical data
☒	☐	Dual use research of concern

Methods

n/a	Involved in the study	
☒	☐	ChIP-seq
☒	☐	Flow cytometry
☒	☐	MRI-based neuroimaging

Antibodies

Antibodies used

Primary antibodies used in this study for immunofluorescence (IF), immunoblotting (IB), or immunoprecipitation (IP), along with their respective antibody dilutions were: anti-IP6K1 (Merck Millipore, HPA040825, C118624, IF 1:500; IB 1:4000); anti-UBF1 (F:9, Santa Cruz, sc-13125, I1509, IF 1:300; IB 1:500; IP 1 µg); anti-FBL (38F3, Santa Cruz, sc-56676; IF 1:50); anti-FLAG (M2, Merck Millipore, F1804, SLBN2445V, IB 1:10,000); anti-GFP (Thermo Fisher Scientific, A11122, 57204A, IB 1:7000; IP 2 µg); anti-myc (9E10, Merck Millipore, M4439, 84292, IB 1:10,000; IP 1 µg); anti-V5 tag (Thermo Fisher Scientific, R960-25, 2129366, IB 1:7000); anti-GAPDH (Merck Millipore, G8795, 104M4751V, IB 1:10,000); anti-α-tubulin (Merck, T9026; IB 1:10,000). HRP-conjugated secondary goat anti-rabbit (4010-05, A4211-ZB66D) and goat anti-mouse (1031-05, D2719-N941C) IgG antibodies for IB (1:10,000) were obtained from Southern Biotech. Fluorophore-conjugated secondary Alexa Fluor 488 (goat anti-mouse IgG; A-11029) and Alexa Fluor 568 antibodies (goat anti-rabbit IgG; A-11011) for IF (1:500) were from Thermo Fisher Scientific.

Validation

As per the manufacturer's website, anti-IP6K1 (Merck Millipore, HPA040825) is validated in the human cell line U-2 OS for IF and human cell line PC-3 for IB; anti-UBF1 (F:9, Santa Cruz, sc-13125) is validated in human cell lines HL-60, mouse cell lines WEHI-231 and NIH/3T3 nuclear extracts for IB and human cell line SW480 for IF; anti-FBL (38F3, Santa Cruz, sc-56676) is validated in the human cell line SH-SY5Y for IF and human cell line Hep G2 whole cell lysates for IB; anti-GAPDH (Merck Millipore, G8795) is validated in several human cell lines including HeLa, A-431 for IB. anti-myc (9E10, Merck Millipore, M4439) is validated in the human cell line HEK293 overexpressing C-terminal c-Myc tagged fusion protein for IF and whole extract of E. coli cells expressing LacZ tagged with c-Myc for IB. anti-FLAG (M2, Merck Millipore, F1804) is validated for the detection of FLAG fusion proteins in mammalian, plant, and bacterial expression systems for IB; anti-V5 tag (Thermo Fisher Scientific, R960-25) is validated in HEK-293 cells transiently overexpressing V5-His-LacZ for IF and whole cell extracts of HEK-293 transiently overexpressing V5-His-LacZ for IB; anti-GFP (Thermo Fisher Scientific, A11122) is validated using H3-GFP construct transfected in HEK-293E cells for IF and GFP-transfected HeLa cell lysates for IF; anti-α-tubulin (Merck, T9026) is validated in many cell line including HeLa, JURKAT, COS7, NIH-3T3 for IB.

Eukaryotic cell lines

Policy information about cell lines and Sex and Gender in Research

Cell line source(s)

HEK293T cells were from ATCC, CRL-3216.
HCT 116 cells used in Fig. 2e were from ATCC, CCL-247.
U-2 OS cells, used in Fig 4b, c and 5g, were from ATCC, HTB-96.
HEK293T cells, used in Fig 4b, f, g, h and i, were from ATCC, CRL-3216, and a gift from Solomon Synder, Johns Hopkins School of Medicine, Baltimore, USA.
HCT116 cells, used in Fig 5 e and f, were a gift from Adolfo Saiardi, University College London, UK.
HEK293T IP6K1-/- cells, used in Fig 5a, c and d, were generated in the lab of Rashna Bhandari, Centre for DNA Fingerprinting and Diagnostics, Hyderabad, India.

Authentication

For data shown in Fig 4b, c, f, g, h, i and 5g, U-2 OS and HEK293T cells were authenticated using STR profiling. For authentication, the total cellular DNA was isolated using our standard protocol. For STR profiling, total ten Genetic loci viz: TH01, D21S11, D5S818, D13S317, D7S820, D16S539, CSF1PO, AMEL, vWA and TPOX were PCR amplified by labelled primers specific to respective loci. The labelled amplicons were resolved by capillary electrophoresis and alleles were called. The sample genotypes were compared with reference STR genotypes available in ATCC®, DSMZ® and Biosample databases to authenticate sample identity and check for cross contamination. The remaining cell lines were not authenticated.

Mycoplasma contamination

All cell-lines tested negative by PCR.

Commonly misidentified lines
(See ICLAC register)

None of the cell lines used in this study are listed as commonly misidentified lines.
