# Supplementary material for: Extensive protein pyrophosphorylation revealed in human cell lines
Source: Nat Chem Biol. 2024 Apr 25;20(10):1305–16. doi: 10.1038/s41589-024-01613-5 (PMC11427299; doi:10.1038/s41589-024-01613-5)
Supplement: Supplementary file 10 — Unprocessed blots and gels. [file 41589_2024_1613_MOESM10_ESM.pdf]

Uncropped blots for Figure 4

HEK293T

Figure 4e

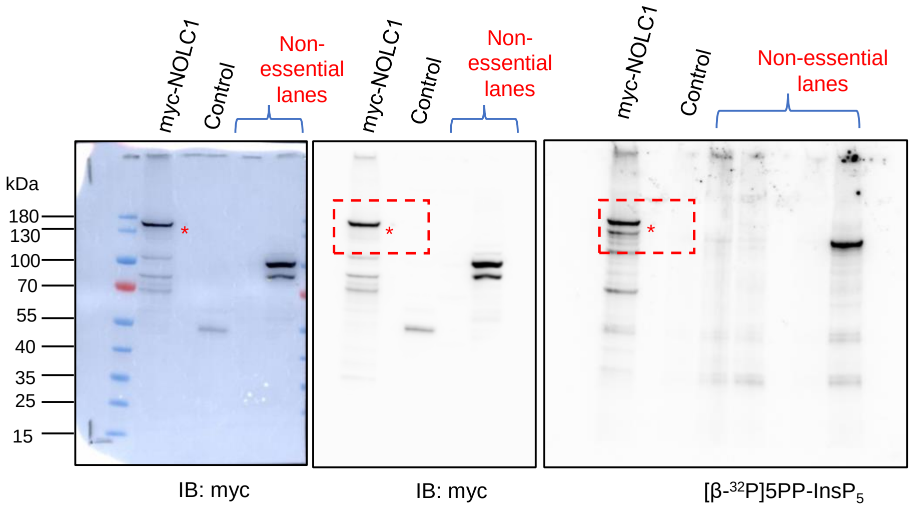

Merge image: Chemiluminescence image overlayed by brightfield image

Chemiluminescence image

Autoradiogram: Equal level adjustment was done on the autoradiogram for better visualisation

Figure 4f

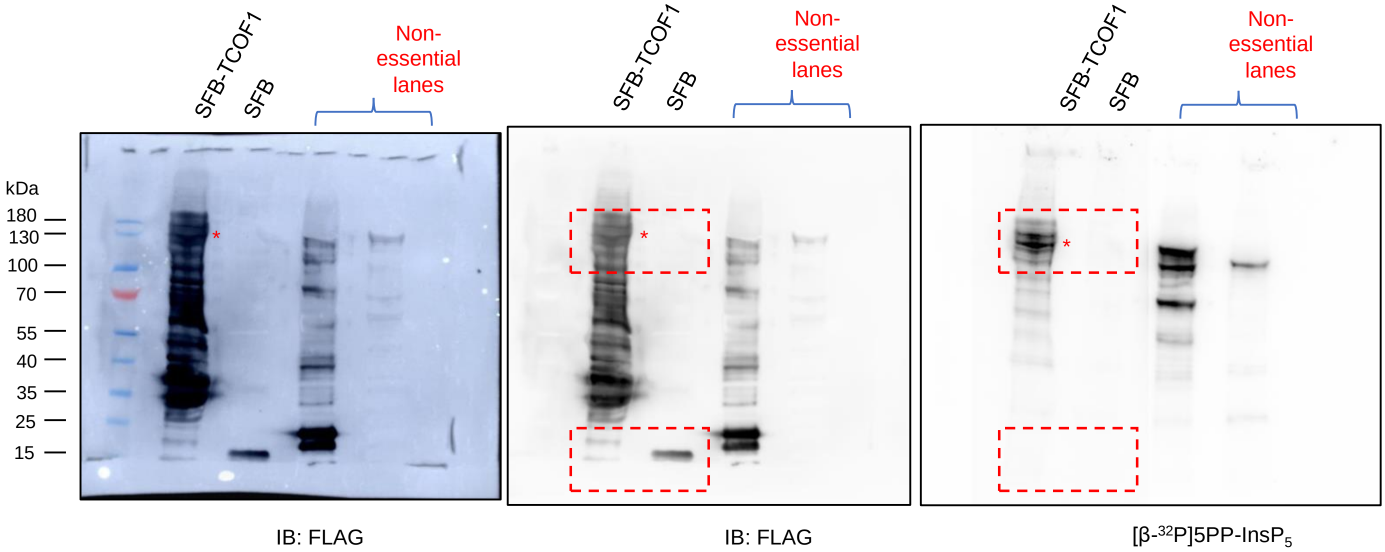

Merge image: Chemiluminescence image overlayed by brightfield image

Chemiluminescence image

Autoradiogram: Equal level adjustment was done SFB-TCOF1 and pcDNA-SFB lane on the autoradiogram for better visualisation

* indicates specific bands

Figure 4g

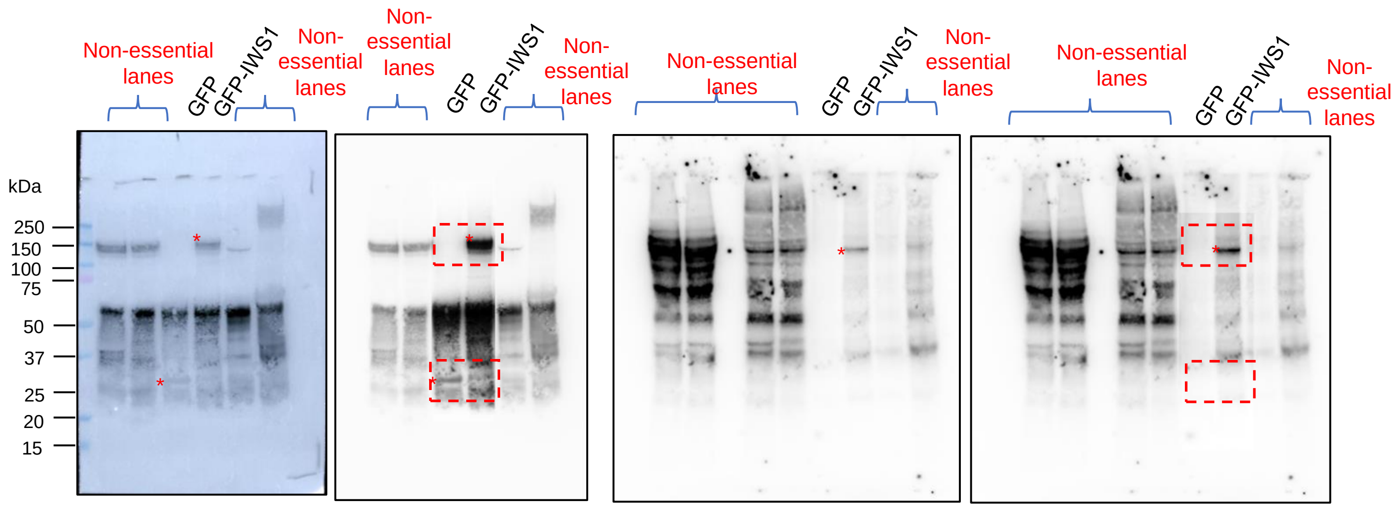

IB: GFP

IB: GFP

[β-^{32}P]5PP-InsP_5

[β-^{32}P]5PP-InsP_5

Merge image: Chemiluminescence image overlayed by brightfield image

Chemiluminescence image: Equal level adjustment was done on GFP and GFP-IWS1 lane

Autoradiogram: Equal level adjustment was done on the autoradiogram for better visualisation

Autoradiogram: Equal level adjustment was done on GFP and GFP-IWS1 lane

Figure 4h

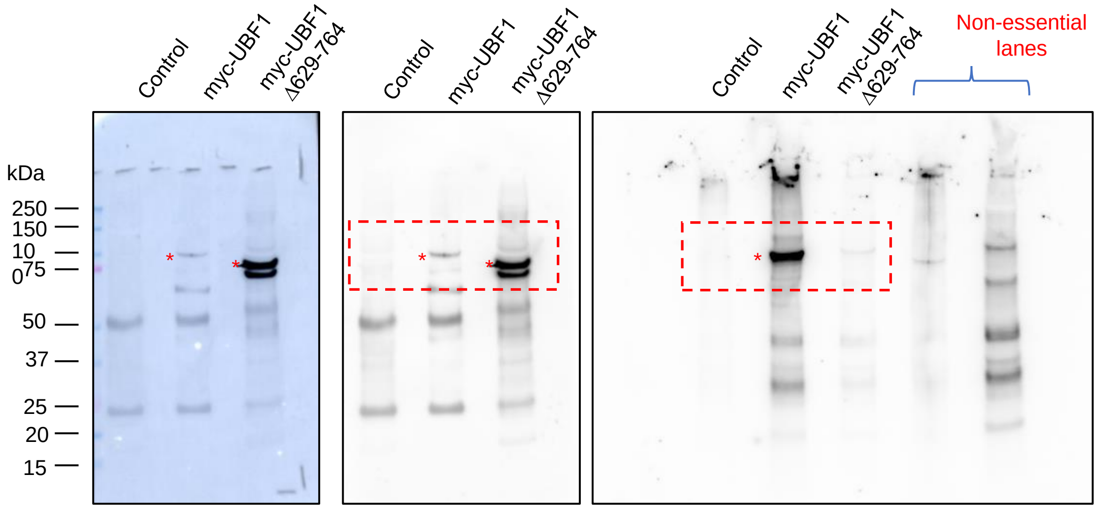

IB: myc

IB: myc

[β-^{32}P]5PP-InsP_5

Merge image: Chemiluminescence image overlayed by brightfield image

Chemiluminescence image: autoleveled for better visualisation

Autoradiogram: Equal level adjustment was done on the autoradiogram for better visualisation

* indicates specific bands
