# Supplementary material for: Extensive protein pyrophosphorylation revealed in human cell lines
Source: Nat Chem Biol. 2024 Apr 25;20(10):1305–16. doi: 10.1038/s41589-024-01613-5 (PMC11427299; doi:10.1038/s41589-024-01613-5)
Supplement: Supplementary file 12 — Unprocessed blots and gels. [file 41589_2024_1613_MOESM12_ESM.pdf]

Uncropped blots for Figure 5

Figure 5a

HEK293T *IP6K1*$^{-/-}$

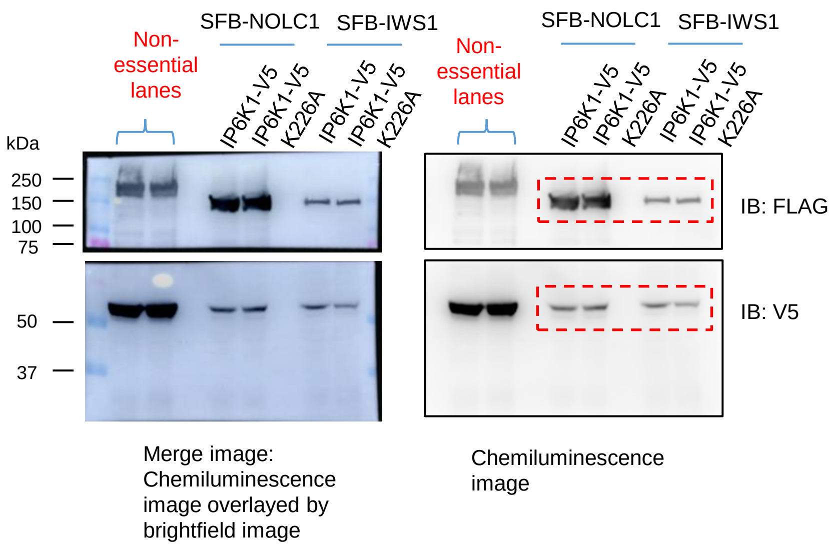

HEK293T *IP6K1*$^{-/-}$

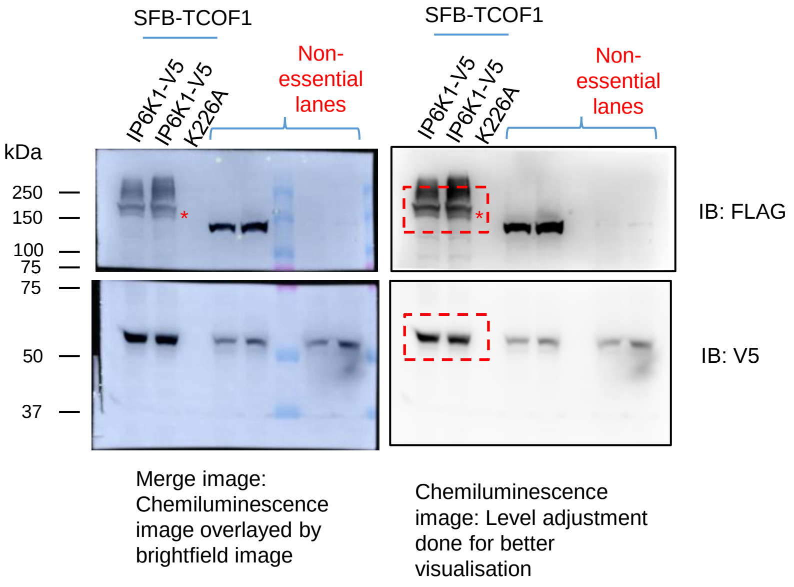

* indicates specific bands

Figure 5c_Replicate 1_Representative blot

HEK293T IP6K1$^{-/-}$
IP: myc

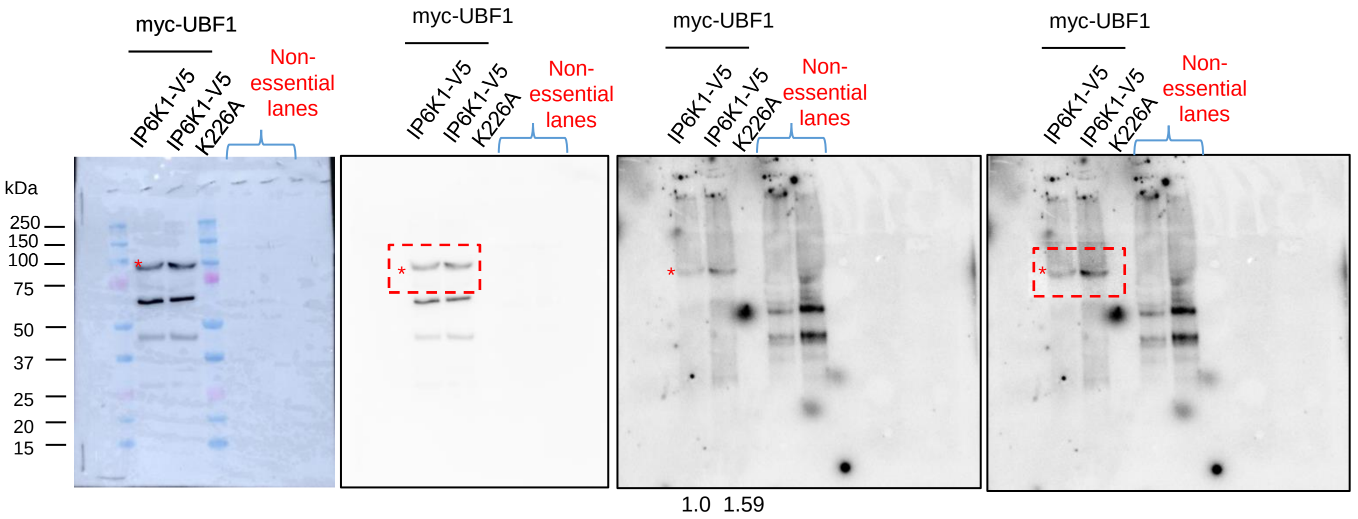

IB: myc
Merge image: Chemiluminescence image overlayed by brightfield image

IB: myc
Chemiluminescence image

[β-^{32}P]5PP-InsP$_5$
Autoradiogram: Equal level adjustment was done on the autoradiogram for better visualisation

[β-^{32}P]5PP-InsP$_5$
Autoradiogram: Equal level adjustment was done on Kinase active and kinase dead o/e lanes for better visualisation

HEK293T *IP6K1*$^{-/-}$
IP: myc

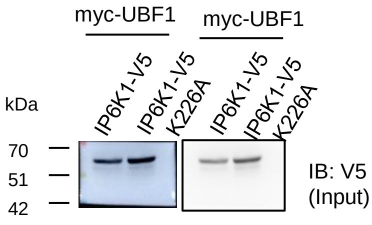

IB: V5 (Input)

Merge image: Chemiluminescence image overlayed by brightfield image

Chemiluminescence image

* indicates specific bands

Figure 5c_Replicate 2

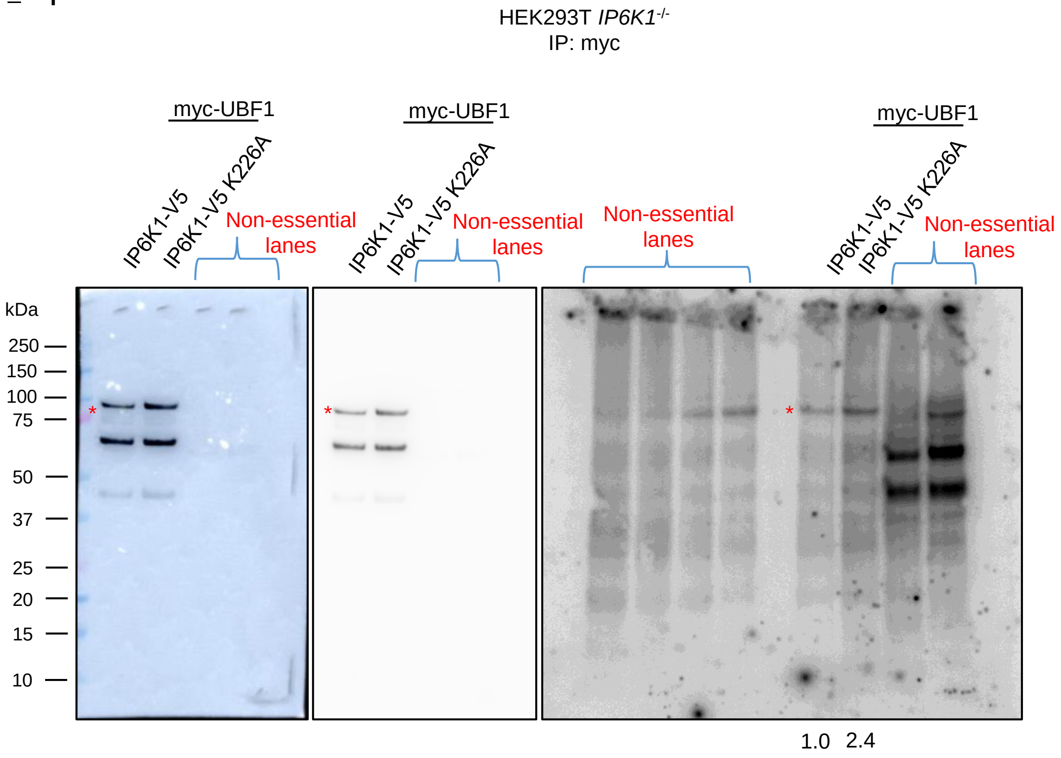

IB: myc
Merge image: Chemiluminescence image overlayed by brightfield image

IB: myc
Chemiluminescence image

[β-^{32}P]5PP-InsP_5
Autoradiogram: Equal level adjustment was done on the autoradiogram for better visualisation

Figure 5c_Replicate 3

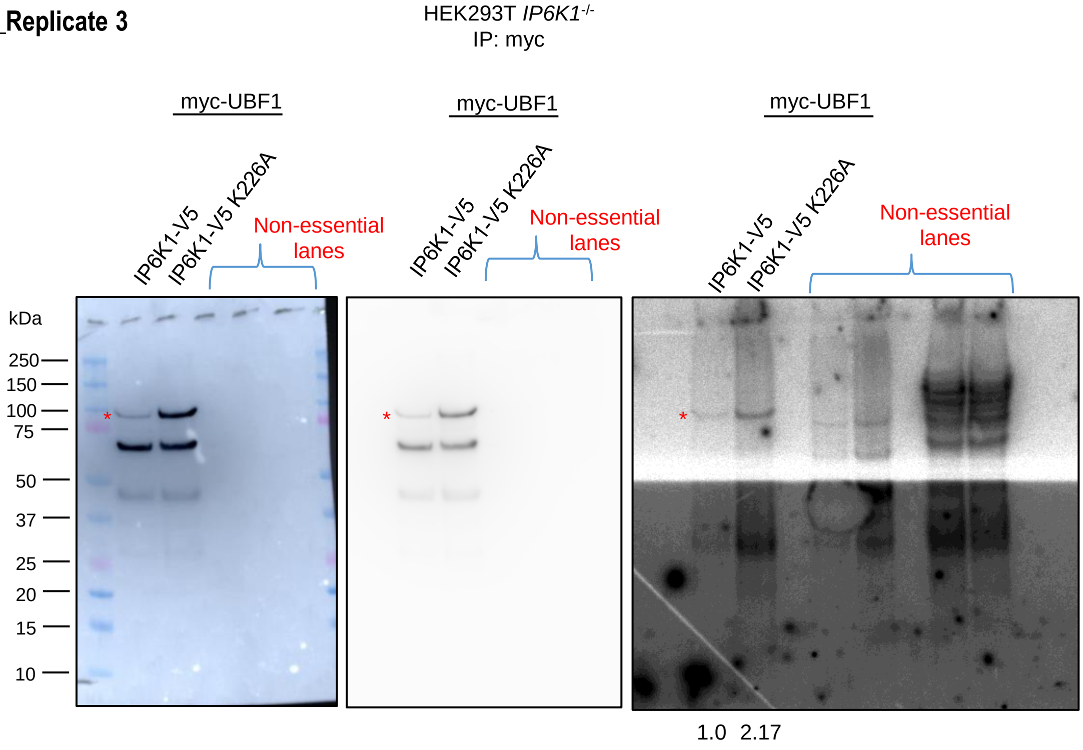

IB: myc
Merge image: Chemiluminescence image overlayed by brightfield image

IB: myc
Chemiluminescence image

[β-^{32}P]5PP-InsP_5
Autoradiogram: Equal level adjustment was done on the autoradiogram for better visualisation

* indicates specific bands

Figure 5d_Replicate 1_Representative blot

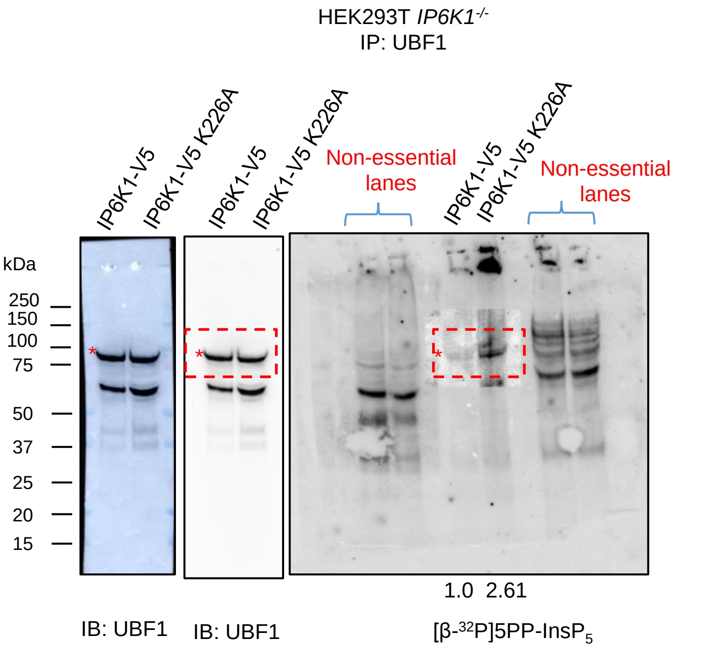

Merge image: Chemiluminescence image overlayed by brightfield image

Chemiluminescence image

Autoradiogram: Equal level adjustment was done on kinase active and kinase dead o/e lanes for better visualisation

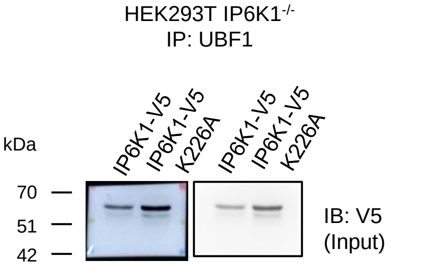

Merge image: Chemiluminescence image overlayed by brightfield image

Chemiluminescence image

Figure 5d_Replicate 2

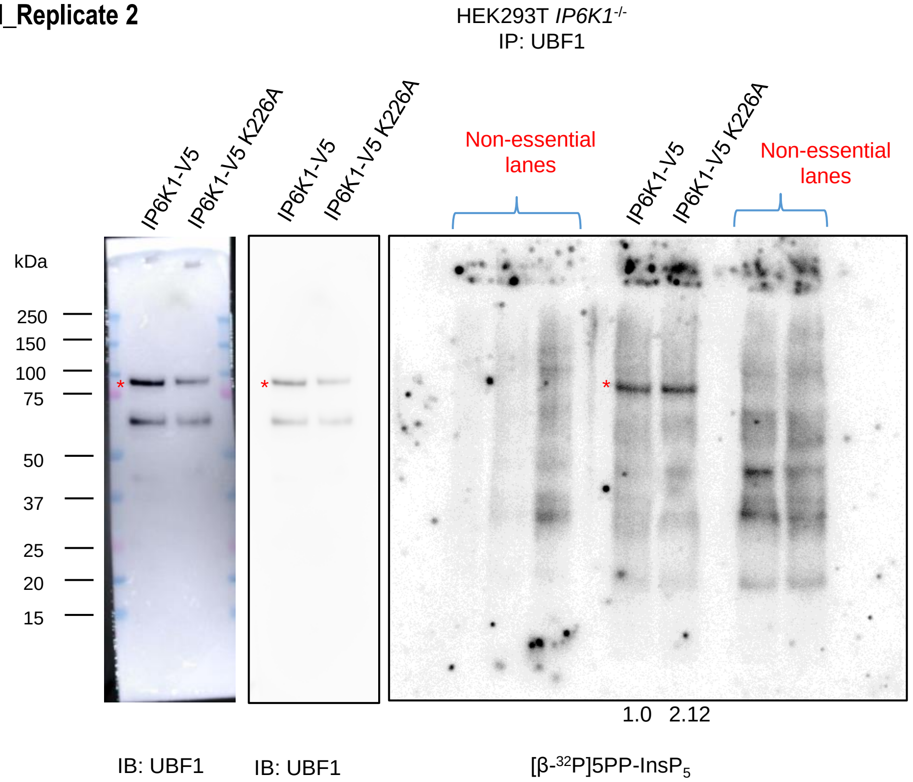

Merge image: Chemiluminescence image overlayed by brightfield image

Chemiluminescence image

Autoradiogram: Equal level adjustment was done on kinase active and kinase dead o/e lanes for better visualisation

* indicates specific bands

Figure 5d_Replicate 3

HEK293T *IP6K1*$^{-/-}$
IP: UBF1

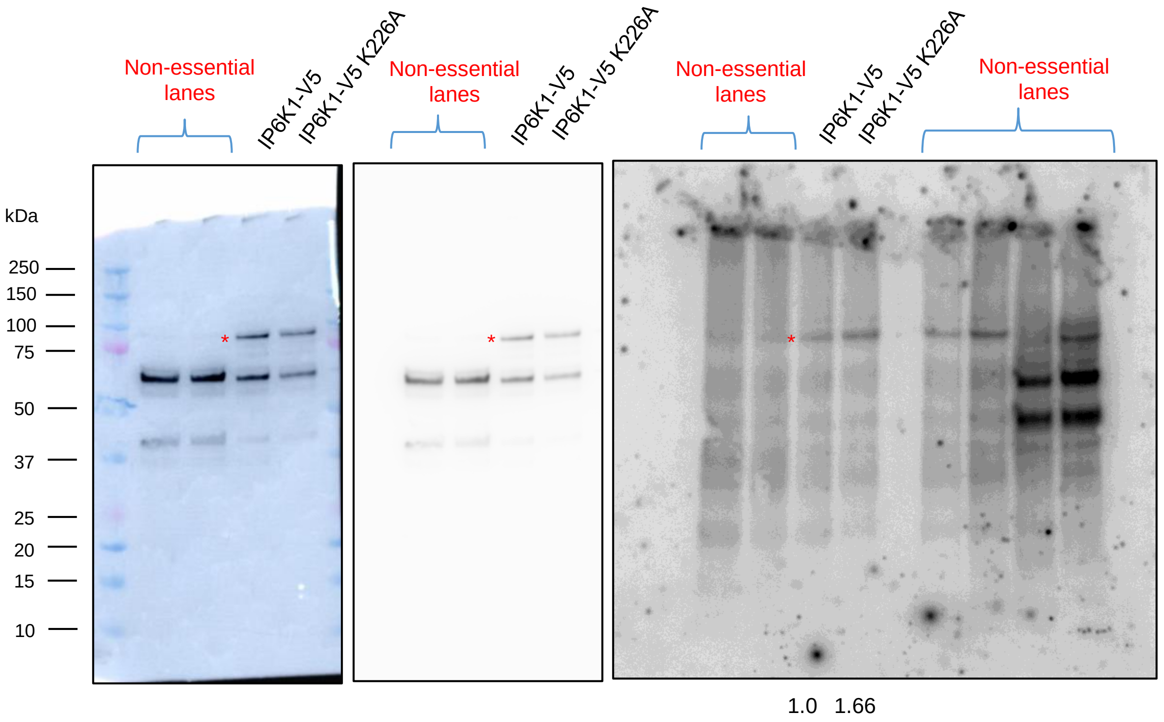

IB: UBF1

Merge image: Chemiluminescence image overlayed by brightfield image

IB: UBF1

Chemilumin-escence image

[β-^{32}P]5PP-InsP$_5$

Autoradiogram: Equal level adjustment was done on kinase active and kinase dead o/e lanes for better visualisation

Figure 5d_Replicate 4

HEK293T *IP6K1*$^{-/-}$
IP: UBF1

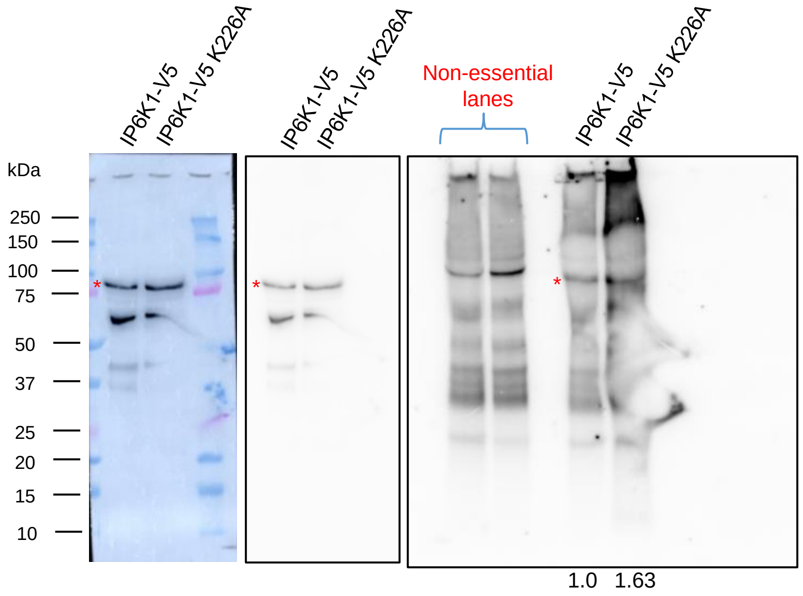

IB: UBF1

Merge image: Chemiluminescence image overlayed by brightfield image

IB: UBF1

Chemilumin-escence image

[β-^{32}P]5PP-InsP$_5$

Autoradiogram: Equal level adjustment was done on kinase active and kinase dead o/e lanes for better visualisation

* indicates specific bands
