# Supplementary material for: Extensive protein pyrophosphorylation revealed in human cell lines
Source: Nat Chem Biol. 2024 Apr 25;20(10):1305–16. doi: 10.1038/s41589-024-01613-5 (PMC11427299; doi:10.1038/s41589-024-01613-5)
Supplement: Supplementary file 14 — Unprocessed blots and gels. [file 41589_2024_1613_MOESM14_ESM.pdf]

Uncropped blots for Extended data Figure 5

Extended data Figure 5a

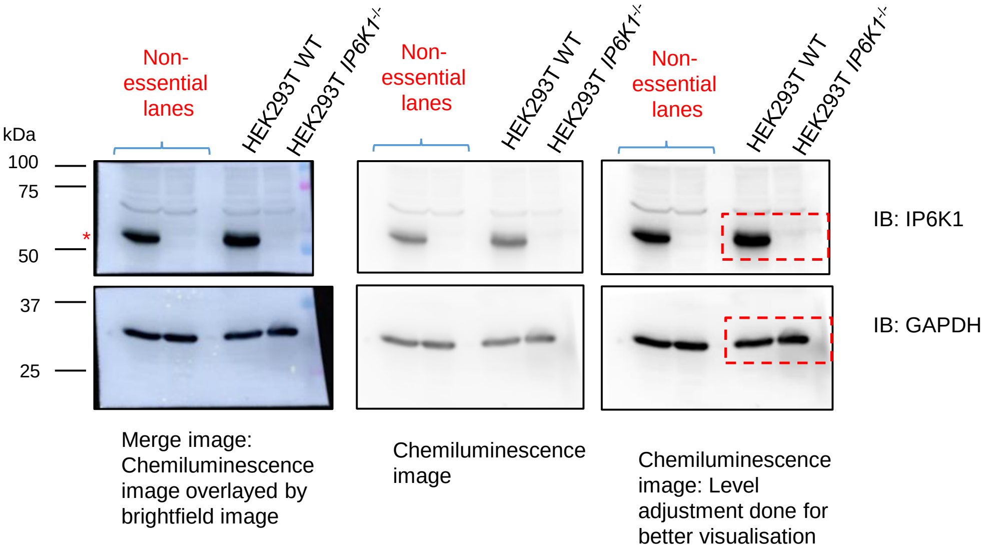

Extended Fig 5c_Replicate 1

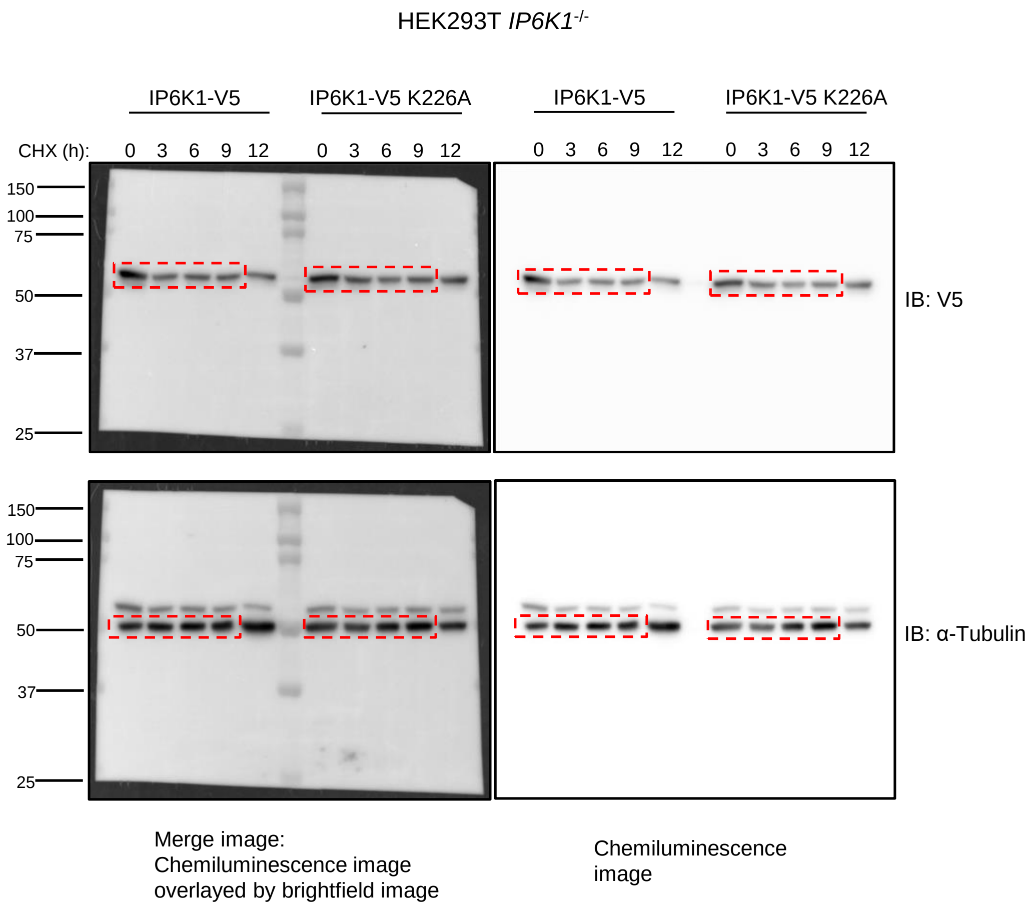

Extended Fig 5c_Replicate 2

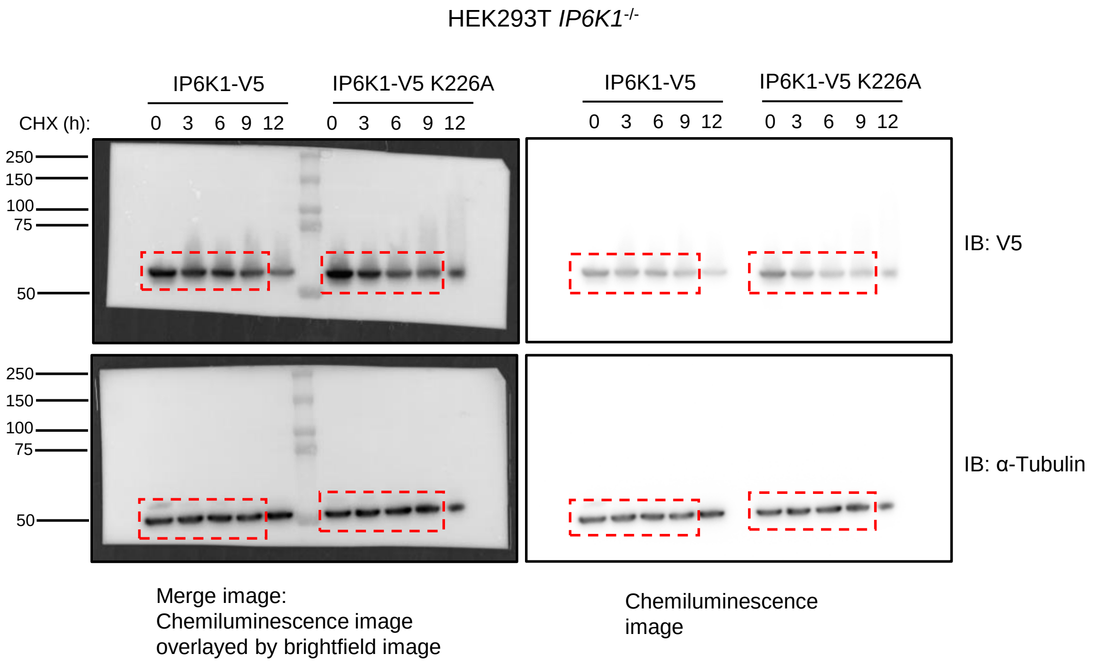

Extended Fig 5c_Replicate 3

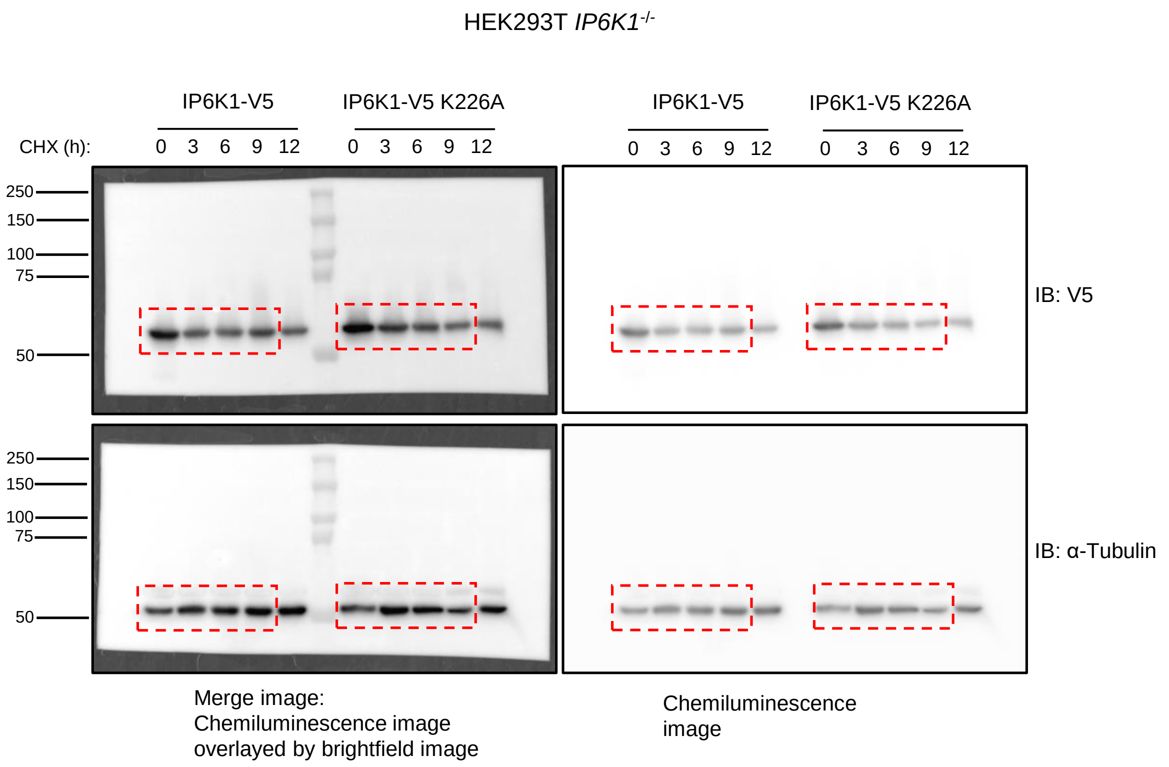

Extended Fig 5c_Replicate 4

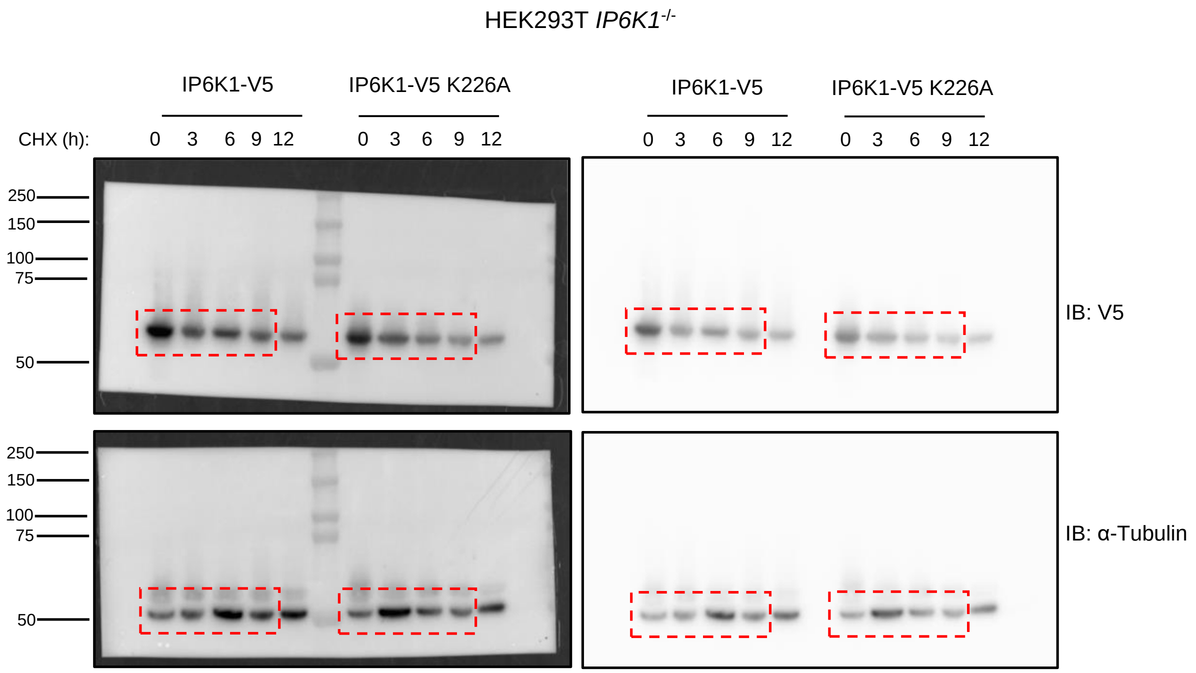

Merge image:
Chemiluminescence image
overlayed by brightfield image

Chemiluminescence
image

Extended Fig 5c_Replicate 5_Representative blot

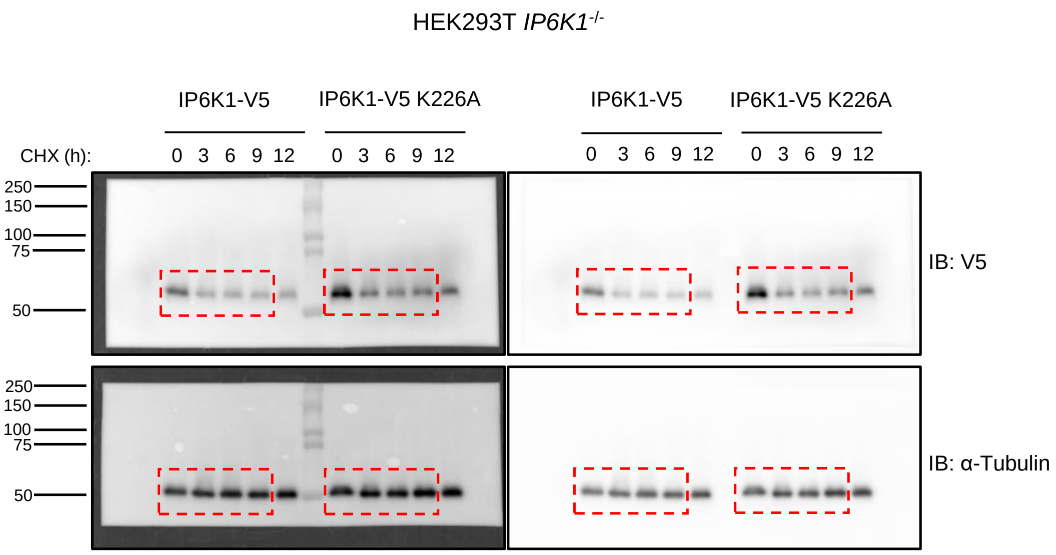

Merge image:
Chemiluminescence image
overlayed by brightfield image

Chemiluminescence
image
